# Emergent Superconductivity and Nonreciprocal Transport in a van der Waals Dirac Semimetal/Antiferromagnet Heterostructure

**DOI:** 10.1021/acs.nanolett.6c01763

**Published:** 2026-08-25

**Authors:** Saurav Islam, Max Stanley, Anthony Richardella, Seungjun Lee, Kalana D. Halanayake, Sandra Santhosh, Danielle Reifsnyder Hickey, Tony Low, Nitin Samarth

**Affiliations:** † Department of Physics, 8082 The Pennsylvania State University, University Park, Pennsylvania 16802, United States; ‡ Materials Research Institute, 8082 The Pennsylvania State University, University Park, Pennsylvania 16802, United States; § Department of Electrical and Computer Engineering, University of Minnesota, Minneapolis, Minnesota 55455, United States; ∥ Department of Applied Physics, Kyung Hee University, Yongin 17104, Republic of Korea; ⊥ Department of Chemistry, 8082 The Pennsylvania State University, University Park, Pennsylvania 16802, United States; # Department of Materials Science and Engineering, 8082 The Pennsylvania State University, University Park, Pennsylvania 16802, United States; 8 School of Physics and Astronomy, University of Minnesota, Minneapolis, Minnesota 55455, United States

**Keywords:** Dirac semimetal, molecular beam epitaxy, antiferromagnet, superconductivity, nonreciprocal transport

## Abstract

We investigate emergent superconductivity and nonreciprocal transport (magnetochiral anisotropy and superconducting diode effect) at the heterointerface of two nonsuperconducting van der Waals (vdW) materials, Dirac semimetal ZrTe_2_ and antiferromagnetic iron chalcogenide FeTe, grown using molecular beam epitaxy. We show from electrical transport measurements that two-dimensional (2D) superconductivity arises at the heterointerface below a critical temperature (*T*_c_) of ∼10 K. In the superconducting transition region, nonreciprocal transport, characterized by magneto-chiral anisotropy, exhibits a magnitude comparable to that observed in topological insulators and is enhanced by a factor of 3 when the heterostructure is capped with a 2D vdW ferromagnet (CrTe_2_). Below *T*_c_, the superconducting diode effect exhibits an efficiency of 29%. With strong spin–orbit coupling in ZrTe_2_, these epitaxial heterostructures provide an attractive epitaxial vdW platform for exploring unconventional superconductivity in Dirac semimetals and for developing nonreciprocal devices for superconducting electronics.

## Graphical abstract

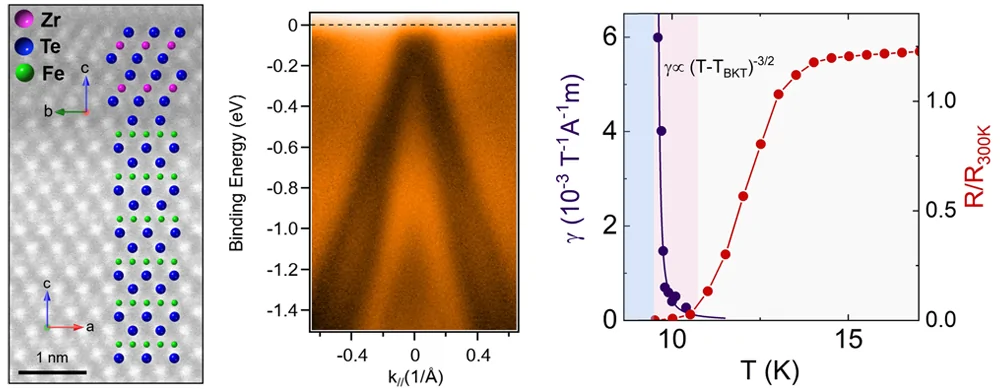

Topological materials interfaced with superconductivity have attracted a significant amount of interest as potential platforms for quantum applications, starting with proposals involving topological insulators^1,2^ and later topological semimetals.^3,4^ Dirac semimetals (DSMs)^5–11^ are particularly appealing within this context for two reasons. First, the nontrivial topology of the bands in the normal state, comprised of Dirac points and surface Fermi loops, is predicted to result in bulk point nodes and surface Majorana modes in the superconducting state, potentially leading to unconventional pairing in some materials.^4^ Second, combining a DSM simultaneously with superconductivity and ferromagnetism could provide a platform for realizing monopole superconductivity.^12–14^ Finally, such hybrid magnetic/topological/superconductor heterostructures that combine broken inversion and time-reversal symmetry with strong spin–orbit coupling provide the necessary ingredients for designing high-efficiency nonreciprocal devices (such as field-free Josephson diodes) for superconducting electronics.^15,16^ Experimental studies of inducing superconductivity in DSMs have largely centered on the canonical material, Cd_3_As_2_, using mesoscopic point contacts^17,18^ or pressure^19^ in bulk crystals, and the proximity effect in nanoplates (≈100 nm)^20–22^ and thin films.^23–25^ Despite these concerted efforts, a materials platform that enables the induction of superconductivity in a well-controlled heterostructure geometry has remained elusive, motivating the continued exploration of alternate strategies to introduce superconductivity into a DSM.

Heterostructures that interface FeTe with other Te-based materials provide an attractive opportunity in this context.^26–36^ Until very recently, the prevailing view considered FeTe as an antiferromagnet (AFM) in its stoichiometric phase, making a transition to a superconducting phase for only nonstoichiometric conditions or under pressure; however, defect-free FeTe has recently been shown to be intrinsically superconducting.^37^ Here, we realize emergent 2D superconductivity and nonreciprocal transport (including an efficient superconducting diode effect) by combining FeTe with van der Waals (vdW) DSM ZrTe_2_ ^38^ and a hybrid vdW DSM/FM heterostructure (ZrTe_2_/CrTe_2_).^39^

We demonstrate the emergence of superconductivity and nonreciprocal transport in these hybrids via magneto-resistance measurements in the linear and nonlinear response regimes as a function of temperature, magnetic field, and current density. Analysis of the current–voltage characteristic (IVC) reveals the emergence of two-dimensional (2D) superconductivity below a *T*_c_ of ∼10 K. Second harmonic electrical transport measurements with an in-plane magnetic field demonstrate nonreciprocal transport, characterized by a magneto-chiral anisotropy coefficient with a magnitude similar to the magnitude of that found in topological insulator/FeTe heterostructures.^27^ The latter study attributed the pronounced nonreciprocal transport to the spin-momentum correlation in helical Dirac surface states; our observation of similar behavior in a DSM with spin-degenerate bulk Dirac bands shows that this is not essential. When the FeTe/ZrTe_2_ heterostructure is capped with a 2D vdW ferromagnet (CrTe_2_), this figure of merit is remarkably enhanced 3-fold, akin to the behavior found in heterostructures that directly interface CrTe_2_ with FeTe,^36^ although the 2D FM here is separated from the FeTe interface by a DSM that is several nanometers thick. In particular, when these hybrids are fully superconducting, we demonstrate a superconducting diode effect with efficiency as high as 29%. Our results demonstrate a new platform that allows systematic manipulation of the interplay of topology, superconductivity, and magnetism at conveniently accessible temperatures (*T* ≥ 4.2 K).

ZrTe_2_ has a layered crystal structure with Zr atoms sandwiched between Te atoms that are stacked along the *c*-axis (Figure 1a).^38,39^ We refer to ZrTe_2_ film thickness in terms of the unit cell (UC) thickness along the *c*-axis. Each unit cell of FeTe is composed of one layer of Fe atoms sandwiched by two layers of Te atoms (Figure 1b).^40,41^ Prior neutron diffraction studies of bulk crystals of FeTe indicate that the spins of the Fe atoms align diagonally across the Fe–Fe square lattice, giving rise to a bicollinear AFM, accompanied by a tetragonal to monoclinic structural phase transition. The FeTe/ZrTe_2_ heterostructures are grown on SrTiO_3_ (100) substrates by molecular beam epitaxy (MBE) (see the Supporting Information^42^).

**Figure 1:**
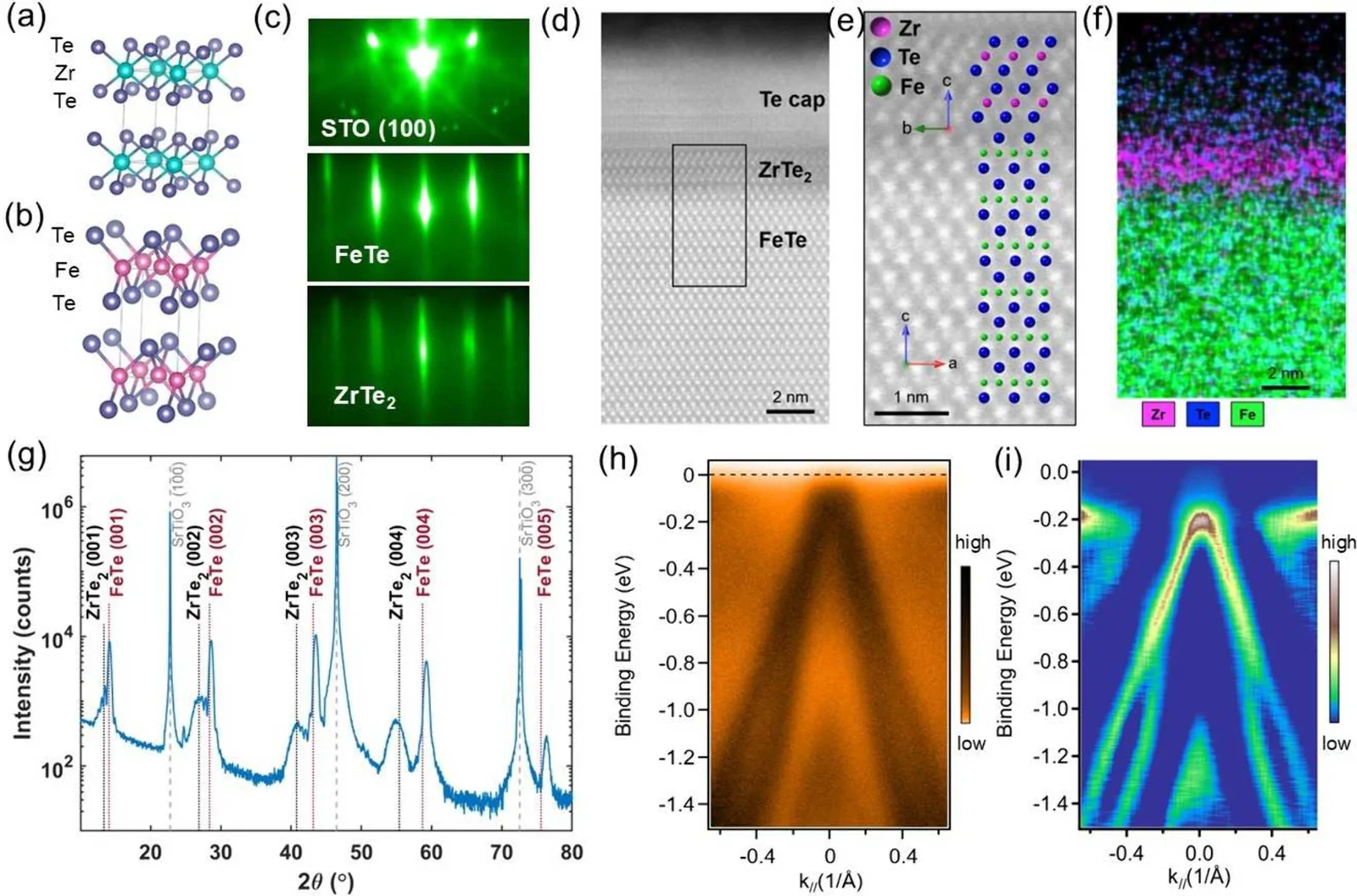
(a) Crystal structure of ZrTe_2_. (b) Crystal structure of FeTe. (c) RHEED images captured during different stages of growth: the pregrowth STO substrate (top), after 20 nm growth of FeTe (middle), and after 3 nm growth of ZrTe_2_ (bottom). (d) Cross-sectional HAADF-STEM image showing the atomically sharp interface between ZrTe_2_ and FeTe. (e) Magnified HAADF-STEM image of the boxed region in panel d. (f) EDX map from a region similar to that shown in panel d. (g) XRD 2θ – ω scans of a ZrTe_2_ (6 UC)/FeTe (35 UC)/SrTiO_3_(100) heterostructure, capped with Te. (h) Angle-resolved photoemission spectra (ARPES) of a 4UC ZrTe_2_/FeTe heterostructure, taken at 300 K along the M̅−Γ̅−M̅ direction using 21.2 eV excitation from a helium lamp. (i) Second derivative of the spectra displayed in panel h.

The reflection high-energy electron diffraction (RHEED) shows streaky patterns consistent with two-dimensional growth and the formation of twinned domains (Figure 1c).^43,44^ Detailed microscopic structural information is obtained using high-angle annular dark-field scanning transmission electron microscopy (HAADF-STEM) imaging of cross-sectional samples. This shows a relatively sharp interface between FeTe and ZrTe_2_ (Figure 1d) (see the Supporting Information^42^). EDX mapping of the cross section reveals the expected composition (Figure 1e). The EDX signal of Zr indicates that ZrTe_2_ layers are located on the FeTe layers. Additionally, the signal from oxygen is also detected in the EDX signal near the ZrTe_2_ layer, resulting from some oxidation of ZrTe_2_ (Figure 1f). We characterize the crystalline quality of the heterostructures *ex situ* using X-ray diffraction (XRD) 2θ – ω scans (Figure 1g) and observe the expected peaks corresponding to SrTiO_3_(100), ZrTe_2_, and FeTe layers (see the Supporting Information^42^). XRD ϕ scans demonstrate that FeTe grows aligned to the SrTiO_3_ substrate while the ZrTe_2_ shows 30° twin domains, consistent with a trigonal material grown on cubic FeTe (see the Supporting Information^42^).

The electronic band structure obtained from *in situ* angle-resolved photoemission spectroscopy (ARPES) for a 4UC ZrTe_2_/FeTe hybrid is shown in Figure 1h (see the Supporting Information^42^). The corresponding second-derivative spectra are shown in Figure 1i. The ARPES data clearly demonstrate linearly dispersed DSM bands, as observed in prior studies of ZrTe_2_ grown on other substrates and buffer layers,^38,39,45^ with the chemical potential located below the Dirac point, consistent with transport measurements (see the Supporting Information^42^).

We now discuss electrical transport measurements, conducted on mechanically scratched Hall bars with a length of 1 mm and a width of 0.5 mm. Figure 2a shows the temperature variation of the sample resistance for 2 UC, 6 UC, and 8 UC ZrTe_2_ and 1 UC CrTe_2_/6 UC ZrTe_2_ grown on 35 UC FeTe. All of these samples show a transition from a normal metal to a superconducting state with a minimum resistance smaller than our measurement limits (*R* ≲ 1 mΩ), except for the 2 UC sample whose resistance does not fully vanish at 2 K. We attribute the latter to inhomogeneity and possibly incomplete coverage of the FeTe with ZrTe_2_ over the scale of the Hall bar device. We observe a distinctive resistance peak around 60 K in all samples. This is more pronounced in the 2 UC sample and broadens as we increase ZrTe_2_ thickness. This feature is consistent with critical spin scattering at the Néel temperature of FeTe as it transitions from a paramagnet to an AFM.^46,47^ In light of recent insights about the tension between antiferromagnetism and superconductivity,^37^ we hypothesize that our as-grown FeTe films likely have Fe interstitials, resulting in AFM order. The transition to a superconducting phase then likely occurs only near the interface where Fe interstitials are removed during the overgrowth of ZrTe_2_. Proving this hypothesis will require additional measurements such as more careful HRTEM or cross-sectional scanning tunneling microscopy. We also observe an excess resistance anomaly before the superconducting transition; we attribute this to nonequilibrium effects at a normal–superconductor interface in the presence of inhomogeneities.^48–50^ The sample-to-sample variability in the position of the resistance peak indicates that its energy scale is not uniquely set by the CrTe_2_-induced exchange field but likely also reflects strain- and stoichiometry-related shifts of the FeTe Néel temperature, with a possible additional contribution from parallel conduction through the CrTe_2_ layer. We also note that CrTe_2_ induces a spin splitting on the order of millielectronvolts throughout the ZrTe_2_ layer, which is comparable to that of the superconducting gap at the ZrTe_2_/FeTe interface (see the Supporting Information^42^).

**Figure 2:**
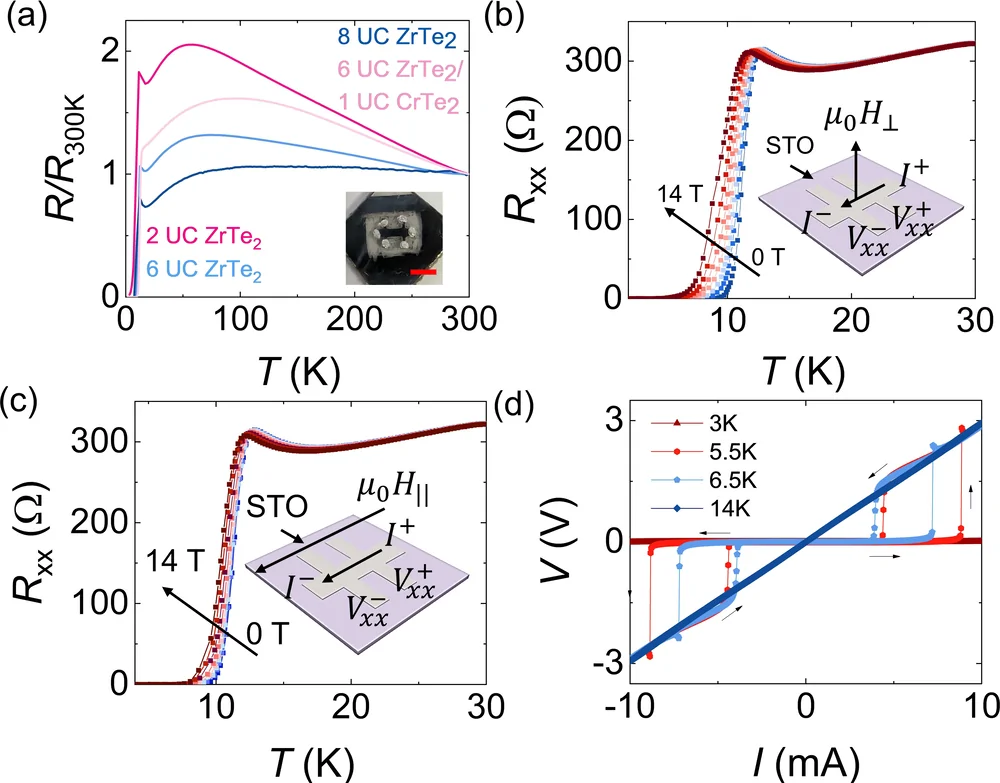
Electrical transport properties. (a) Longitudinal resistance normalized by the 300 K magnitude (*R*/*R*_300K_) vs temperature (*T*) for different films. The inset shows an optical image of a scratched Hall bar device. The scale bar is 1 mm. (b) *R*_*xx*_ vs *T* as a function of magnetic field μ_0_ *H* directed perpendicular to the sample plane. (c) Data similar to those depicted in panel b with the magnetic field in plane and parallel to the current. (d) Current–voltage characteristic vs temperature, showing clear switching and hysteresis.

To gain deeper insights into the nature of the superconducting state, we measured the temperature-dependent resistance in different magnetic fields, applied perpendicular and parallel to the sample plane; this is shown in panels b and c, respectively, of Figure 2 for the 6 UC ZrTe_2_ sample (see the Supporting Information^42^). The general characteristics observed here are superficially similar to the behavior reported in prior studies of emergent superconductivity in FeTe-based heterostructures.^26–36^ The upper critical field is clearly very large (≥14 T) for both in-plane and out-of-plane applied-field directions. The superconducting transition exhibits a pronounced anisotropy with respect to the direction of the applied external magnetic field. The broadening of the transition is significantly weaker when the magnetic field is applied parallel to the interface, compared to that with perpendicular application. We also measure the nonlinear current–voltage characteristic (IVC) of the films close to the transition and find a hysteretic behavior with *T*-dependent switching (*I*_c_) and retrapping (*I*_r_) currents, where |*I*_r_| < |*I*_c_| (Figure 2d), attributed to the formation of hot spots (see the Supporting Information^42^ for the *T* dependence of *I*_c_). We next extract the upper critical field (*H*_c2_) as a function of the reduced temperature (*T*/*T*_c_) for both perpendicular and parallel magnetic-field directions (Figure 3a). We define the transition temperature (*T*_c_) as the temperature at which *R*_*xx*_ decreases to 50% of the value recorded at 30 K. We use the weak coupling BCS theory for 2D superconductors (*T*_c_/*T*_f_ < 0.005, with *T*_f_ being the Fermi temperature^51,52^ ) to fit the critical field versus reduced temperature (*T*/*T*_c_) and extract the zero-temperature coherence length (ξ_0_) and superconducting length (*d*_sc_).^26^ Here, the perpendicular critical-field (*H*_c2,⊥_) dependence is given by

1 $$\mu_{0}H_{c2,⊥}\left(T\right)=\frac{\Phi_{0}}{2\pi {\xi_{0}}^{2}}\left(1-T/T_{c}\right)$$

whereas the parallel critical-field (*H*_c2,∥_) dependence is given by

2 $$\mu_{0}H_{c2,∥}\left(T\right)=\frac{\sqrt{3}\Phi_{0}}{\pi \xi_{0}d_{\mathrm{sc}}}{\left(1-T/T_{c}\right)}^{1/2}$$

where Φ_0_ is the magnetic flux quantum. From reasonable fits to our data (Figure 3a), we estimate ξ_0_ = 1.9 nm and superconducting length *d*_sc_ = 12.9 nm. In all samples studied here, the experimentally extracted in-plane critical field extrapolated to 0 K is twice the value expected theoretically for Pauli pair breaking.^53,54^ The apparently ubiquitous observation of this violation in FeTe-based heterostructures raises interesting questions regarding the pairing symmetry but is beyond the scope of the present work.

**Figure 3:**
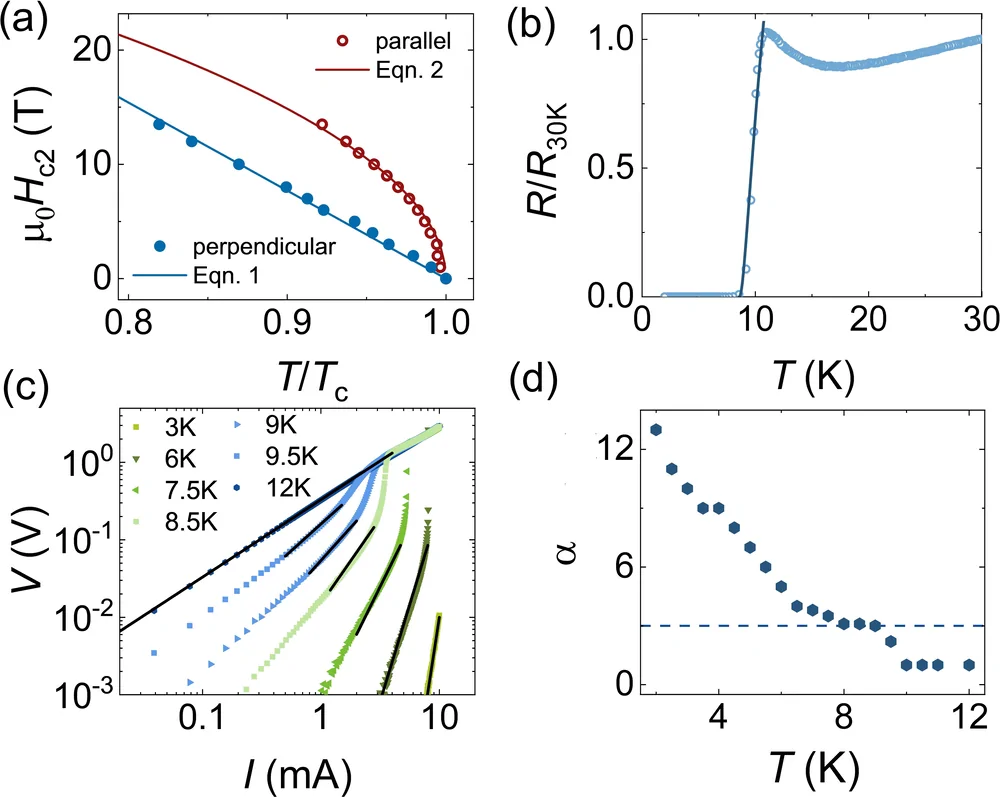
Two-dimensional superconductivity. (a) Upper critical magnetic field (*H*_c2_) as a function of reduced temperature (*T*/*T*_c_). The solid black and blue lines are fits to the data according to eqs 1 and 2, respectively. (b) *R*/*R*_30K_ vs *T* for the 6 UC sample. The solid line is fit to the data according to the Halperin–Nelson equation. (c) Current–voltage characteristic plotted on a log–log scale. Solid lines are fits to the data according to *V* ∝ *I* ^α^. (d) α as a function of temperature, showing a rapid increase below the transition.

Since superconductivity in our heterostructures is assumed to be 2D in nature, arising at the interface with FeTe, as hypothesized in other FeTe-based heterostructures,^26,28,34^ we analyze the temperature dependence of sample resistance according to the Berezinskii–Kosterlitz–Thouless (BKT) model for 2D superconductors. This is best described using the Halperin–Nelson equation, where $R\left(T\right)\propto R_{0}\exp^{\left(-b/{\left(T-T_{BKT}\right)}^{1/2}\right)}$ . Here, *R*_0_ and *b* are material-specific constants.^26,55–58^ Figure 3b shows that this model works reasonably well for the 6 UC samples, generating a *T*_BKT_ of 9.6 K. Within the BKT framework, the IV relationship follows a power-law dependence (*V* ∝ *I* ^α^), where α depends on the temperature.^59,60^ α = 3 is a hallmark of the BKT transition arising due to the onset of free vortex–antivortex motion. We fit the IVC within the BKT framework (Figure 3c) and show the corresponding magnitude of α in Figure 3d. Above the transition, the IVC is linear while the value of α increases rapidly below *T*_c_ to 13 at 2 K. At 9.5 K, α = 3, which corresponds to *T*_BKT_. This is in excellent agreement with the analysis of the *R* versus *T* data, providing strong support for the 2D nature of superconductivity in these samples.

We next examine nonreciprocal transport to explore the interaction between superconductivity and topological states. When an in-plane magnetic field is applied (Figure 4a), longitudinal voltage *V*_*xx*_ under broken inversion symmetry is phenomenologically given by

3 $$V_{xx}=R^{\omega }I\left(1+\gamma \mu_{0}\left(Hẑ\right)\times I\right)$$

where *R* ^ω^ is the first-harmonic longitudinal resistance and γ is the magneto-chiral anisotropy coefficient that quantifies the strength of the nonreciprocal charge transport. When the current is perpendicular to the applied in-plane field, eq 3 reduces to *V*_*xx*_ = *R* ^ω^ *I* + *γμ*_0_ *R* ^ω^ *HI* ^2^. We use the second term to extract γ by measuring the second harmonic signal under an applied ac current due to its quadratic nature, where $R^{2\omega }=\frac{R^{\omega }}{\sqrt{2}}\gamma \mu_{0}HI$ . Experimentally, *R* ^2ω^ is measured as a function of magnetic field at different temperatures (see the Supporting Information^42^). γ can be extracted from the slope of *R* ^2ω^/*R* ^ω^ versus *H*. We extract γ as a function of temperature (Figure 4b) and divide the response into three regions: normal, intermediate, and superconducting. In the superconducting region, both *R* ^2ω^ and *R* ^ω^ vanish, whereas in the normal region, *R* ^2ω^ becomes negligible. In the intermediate region where *T*_BKT_ < *T* < *T*_c,onset_, the extracted value of γ appears to diverge with a functional form $\propto {\left(T-T_{\mathrm{BKT}}\right)}^{-3/2}$ . In the 6 UC sample, the magnitude of γ, normalized to current density, is comparable to that observed in Bi_2_Te_3_/FeTe hybrids with a maximum of 6 A^–1^ T^–1^ m. The enhancement of the magneto-chiral anisotropy arises due to a shift of the energy scale from the Fermi energy (*E*_f_) to the superconducting gap (Δ_SC_), which is smaller.^61,62^ We also observe a 3-fold enhancement in the magnitude of γ when a monolayer of CrTe_2_, a 2D vdW ferromagnet with perpendicular magnetic anisotropy, is grown on top of the STO/FeTe/ZrTe_2_ (6 UC) heterostructure (Figure 4b) (see the Supporting Information^42^). We are tempted to attribute this to the removal of time-reversal symmetry, as in prior studies of ferromagnets directly interfaced with FeTe,^36^ but note that the ferromagnetic layer in our samples is ∼4 nm from the FeTe interface where superconductivity presumably resides. Another aspect of nonreciprocity is the superconducting diode effect, which manifests as an asymmetry in the critical current in the positive and negative current branches^64,65^ (Figure 4c and the Supporting Information^42^). Figure 4d shows the difference between the magnitude of the critical current between positive and negative sweep directions, $\Delta I_{c}=I_{c}^{+}-\left|I_{c}^{-}\right|$ , as a function of magnetic field for the STO/FeTe/ZrTe_2_ (6 UC)/CrTe_2_ (1 UC) heterostructure. Here, the midpoint of the *V*–*I* curve is defined as the critical current with $I_{c}^{+}$ and $\left|I_{c}^{-}\right|$ denoting the corresponding values for positive and negative current sweeps, respectively.^64^ While Δ*I*_c_ decreases as μ_0_ *H*_c2,∥_ increases, the efficiency of the diode effect, $\eta =\frac{I_{c}^{+}-\left|I_{c}^{-}\right|}{I_{c}^{+}+\left|I_{c}^{-}\right|}$ , moderately increases with μ_0_ *H* and can be as high as 29%, which is similar to the highest reported values in the literature.^66^ We note here that the Pauli limit violation combined with the high diode efficiency is suggestive of unconventional or finite-momentum pairing in the interfacial superconducting state, but the current measurements do not permit a distinction between a spin–orbit-coupled superconductor with broken inversion and time-reversal symmetry and a genuine finite-momentum pairing state.^67^ We note that we could not reliably extract the critical current for magnetic fields below 3 T over the temperature range investigated; also, corresponding measurements under negative magnetic fields are not available.

**Figure 4:**
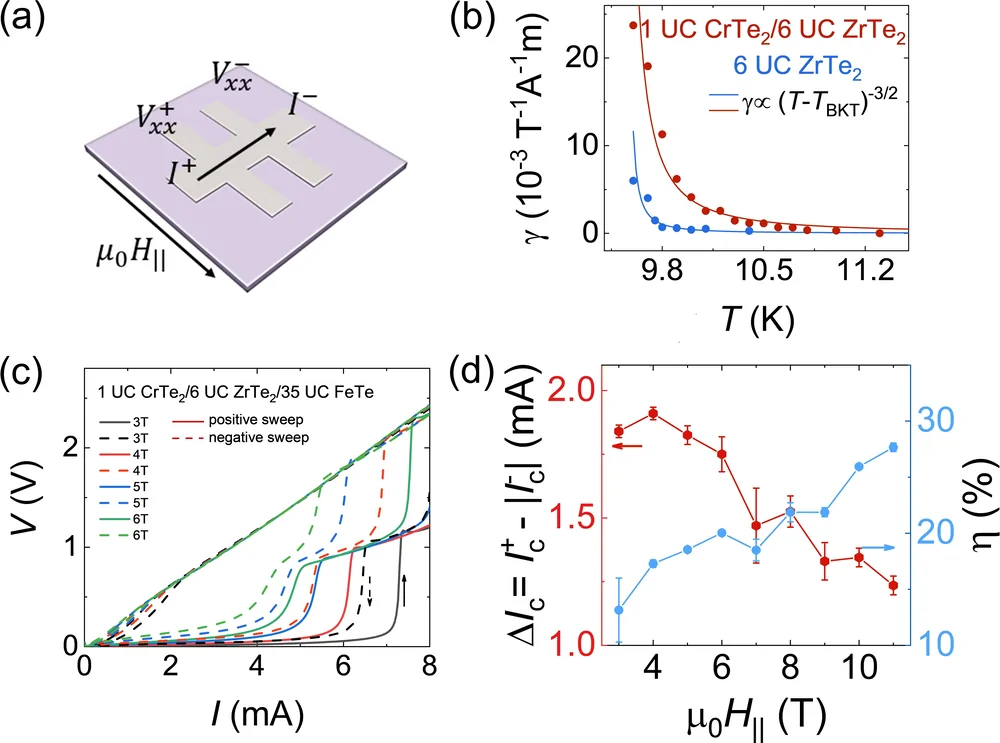
Nonreciprocal charge transport. (a) Schematic of the second harmonic resistance measurement to extract magneto-chiral anisotropy. The current (*I*) and magnetic field (*B*) are in plane and perpendicular to each other. (b) Temperature dependence of magneto-chiral coefficient γ measured at 600 μA. The solid line is fit to the data using the relationship $\gamma \propto {\left(T-T_{BKT}\right)}^{-3/2}$ . (c) Current–voltage characteristics for 1 UC CrTe_2_/6 UC ZrTe_2_/35 UC at representative magnetic fields of 3, 4, 5, and 6 T, showing asymmetry in the critical current between the positive and negative sweep directions. The solid (dashed) lines represent the positive (negative) sweep direction. (d) Difference between the magnitude of the critical current between positive and negative current sweep directions, $\Delta I_{c}=I_{c}^{+}-\left|I_{c}^{-}\right|$ , plotted as a function of magnetic field (left axis). Efficiency $\eta =\frac{I_{c}^{+}-\left|I_{c}^{-}\right|}{I_{c}^{+}+\left|I_{c}^{-}\right|}$ is plotted as a function of magnetic field (right axis).

In the framework discussed by Yuan and Fu,^68^ a superconducting diode effect arises when inversion symmetry breaking and time-reversal symmetry breaking allow odd-in-momentum terms in the Ginzburg–Landau free energy of a current-carrying superconducting condensate. A supercurrent corresponds to a finite condensate momentum *q*, and in a spin–orbit-coupled noncentrosymmetric superconductor, the free energy may contain terms of the form

4 $$F\left(\mathrm{q⃗},\Delta \right)=\left\{t+a_{0}q^{2}-b_{0}\mathrm{q⃗}\cdot \left({\mathrm{h⃗}}_{e\mathrm{ff}}ẑ\right)+b_{1}q^{2}\mathrm{q⃗}\cdot \left({\mathrm{h⃗}}_{e\mathrm{ff}}ẑ\right)\right\}\left|\Delta {|}^{2}+\frac{\beta }{2}\right|\Delta {|}^{4}$$

where ẑ is the polar axis of the ZrTe_2_/FeTe interface, ${\mathrm{h⃗}}_{e\mathrm{ff}}$ is the effective time-reversal-breaking field, and Δ is the superconducting order parameter. The term $q^{2}\mathrm{q⃗}\cdot \left({\mathrm{h⃗}}_{e\mathrm{ff}}ẑ\right)$ is odd in *q* and skews the depairing free energy for positive and negative current directions. This produces unequal critical currents in opposite directions, which is the superconducting diode effect. Thus, the diode response is controlled by not only the existence of inversion breaking but also the magnitude of the effective time-reversal-breaking field acting on the spin–orbit-coupled superconducting condensate. In our heterostructure, the effective field can be written phenomenologically as ${\mathrm{h⃗}}_{e\mathrm{ff}}={\mathrm{h⃗}}_{ex}+{\mathrm{h⃗}}_{prox}$ The first term, ${\mathrm{h⃗}}_{ex}$ , is the ordinary Zeeman coupling to the applied magnetic field. The second term, ${\mathrm{h⃗}}_{prox}$ , represents the exchange-proximity contribution from the CrTe_2_ layer. Thus, the CrTe_2_ cap does not provide inversion symmetry breaking; that is already present at the ZrTe_2_/FeTe interface. Instead, CrTe_2_ provides an additional exchange field that adds to the applied-field Zeeman term. Since the odd-in-*q* terms responsible for asymmetric depairing scale with ${\mathrm{h⃗}}_{e\mathrm{ff}}$ , this additional exchange contribution provides a natural microscopic mechanism for the enhanced superconducting diode response observed after CrTe_2_ capping. To further support this interpretation, we have included first-principles calculations of the proximity effect in the Supporting Information (Figures S13–S15). Specifically, we calculate the spin density in the CrTe_2_/ZrTe_2_ heterostructure and find that the magnetic polarization associated with CrTe_2_ induces a finite spin density in the adjacent ZrTe_2_ layer. This result provides microscopic support for phenomenological exchange-proximity term ${\mathrm{h⃗}}_{prox}$ used above. We further note that although the calculated spin polarization decays rapidly away from the CrTe_2_/ZrTe_2_ interface, this small magnetic proximity gives rise to a millielectronvolt-scale spin splitting throughout the ZrTe_2_ layer (see the Supporting Information^42^). This splitting is not negligible compared to the energy scale of the superconducting gap at the ZrTe_2_/FeTe interface.

The detailed underlying physical origin of interface-induced superconductivity in FeTe-based hybrids remains unresolved,^32^ although a recent report of stoichiometric FeTe being a superconductor suggests that capping as-grown FeTe films with other tellurides can effectively anneal away interstitial defects.^37^ The interplay between antiferromagnetism and superconductivity was investigated in ref 69; the authors observed that antiferromagnetism in FeTe is suppressed in (BiSb)_2_Te_3_/FeTe and Cr-(BiSb)_2_Te_3_/FeTe hybrids yet coexists with superconductivity when they are interfaced with MnBi_2_Te_4_. To gain further insight into the superconductivity observed in our experiments, we perform first-principles calculations based on density functional theory (DFT) (see the Supporting Information for details^42^). As a first step, we investigate the role of the ZrTe_2_ layer by calculating the interfacial charge transfer in ZrTe_2_/FeTe heterostructures (Figure S12c). The results reveal an accumulation of electrons in the ZrTe_2_ layer, indicating hole doping in FeTe near the interface. This finding is further supported by additional DFT calculations for isolated bilayer ZrTe_2_ and FeTe, which show that 2 UC of ZrTe_2_ has a higher work function (4.83 eV) than 2 UC of FeTe (4.25 eV). Next, we examine the relative stability of the bicollinear and stripe antiferromagnetic phases in 2 UC FeTe as a function of hole doping (Figure S12d). In the pristine case, although the bicollinear AFM is the experimental ground state of FeTe, the total energy of the stripe AFM phase is calculated to be lower than that of the bicollinear AFM, consistent with previous theoretical reports.^70^ We note that the relative stabilities of magnetic phases can be sensitive to various computational details. Therefore, in the following discussion, we focus on the trend with hole doping, based on our theoretical results. Interestingly, we find that hole doping stabilizes the bicollinear AFM, suggesting that this phase remains the ground state in the experimentally studied ZrTe_2_/FeTe heterostructure. Therefore, the interfacial superconductivity observed in the ZrTe_2_/FeTe heterostructure may coexist with the bicollinear antiferromagnetic order, similar to previous observations in FeTe/Bi_2_Te_3_ ^26,33^ and FeTe/MnBi_2_Te_4_ ^35^ heterostructures. These results indicate that the primary role of ZrTe_2_ is to induce charge transfer and modulate the magnetic order of FeTe, providing new insights into the origin of the mysterious interfacial superconductivity in FeTe-based heterostructures.

In conclusion, we have demonstrated an epitaxial vdW platform to induce superconductivity in a DSM, ZrTe_2_, while simultaneously interfacing it with an AFM (FeTe) and a 2D vdW FM (CrTe_2_). Through detailed electrical transport measurements, we demonstrate that 2D superconductivity emerges at an interface below a critical temperature of 12 K. This is an unusual superconducting state that supports nonreciprocal transport, as revealed through magneto-chiral anisotropy and the superconducting diode effect. The especially high efficiency (29%) of the latter in hybrid CrTe_2_/ZrTe_2_/FeTe heterostructures promises an attractive wafer-scale vdW platform for superconducting electronics. Although the current transport data reflect the response of the heterostructure, the state of ZrTe_2_ Dirac fermions in the superconducting state remains an open question that needs to be addressed. Beyond the present study, these hybrid epitaxial heterostructures provide a potentially versatile platform for exploring topological superconductivity and its interaction with magnetism.

## Supplementary Material



## Notes

Molecular beam epitaxy of ZrTe_2_/FeTe hybrids, transmission electron microscopy, X-ray diffraction, angle-resolved photoemission spectroscopy, magnetic-field dependence of different samples, temperature dependence of critical current, second-harmonic measurements, electrical properties of CrTe_2_/ZrTe_2_/FeTe hybrids, current–voltage characteristics, DFT calculations, and effect of growth temperature (PDF)
