# Supplementary material for: Emergent Superconductivity and Nonreciprocal Transport in a van der Waals Dirac Semimetal/Antiferromagnet Heterostructure
Source: Nano Lett. 2026 Aug 25;26(35):11671–8. doi: 10.1021/acs.nanolett.6c01763 (PMC13564870; doi:10.1021/acs.nanolett.6c01763)
Supplement: Supplementary file 1 [file nl6c01763_si_001.pdf]

Supplementary Materials: Emergent superconductivity and non-reciprocal transport in a van der Waals Dirac semimetal/antiferromagnet heterostructure

Saurav Islam,[1,2,*] Max Stanley,[1,2] Anthony Richardella,[1,2] Seungjun Lee,[3,4] Kalana D. Halanayake,[5] Sandra Santhosh,[1] Danielle Reifsnyder Hickey,[5,6,2] Tony Low,[7,8] and Nitin Samarth[1,6,2,†]

[1]*Department of Physics, Pennsylvania State University, University Park, Pennsylvania 16802, USA*

[2]*Materials Research Institute, Pennsylvania State University, University Park, Pennsylvania 16802, USA*

[3]*Department of Electrical and Computer Engineering, University of Minnesota, Minneapolis, MN 55455, USA.*

[4]*Department of Applied Physics, Kyung Hee University, Yongin 17104, Republic of Korea*

[5]*Department of Chemistry, Pennsylvania State University, University Park, Pennsylvania 16802, USA*

[6]*Department of Materials Science and Engineering, Pennsylvania State University, University Park, Pennsylvania 16802, USA*

[7]*Department of Electrical and Computer Engineering, University of Minnesota, Minneapolis, MN, 55455, USA*

[8]*School of Physics and Astronomy, University of Minnesota, Minneapolis, MN, 55455, USA*

I. MOLECULAR BEAM EPITAXY OF $ZRTE_2$/FETE HYBRIDS

$ZrTe_2$ has a layered crystal structure, belonging to the space group P$\bar{3}$m1. The arrangement of atoms within each layer exhibits a hexagonal pattern while each Zr atom is surrounded by six Te atoms in an octahedral configuration with $a = b = 3.95 \mathring{A}$ (in-plane), and $c = 6.63 \mathring{A}$ (out-of-plane). The lattice constants of FeTe at room temperature are $a = b = 3.82$ Å (in-plane) and $c = 6.25$ Å (out-of-plane).

The $ZrTe_2$/FeTe heterostructures were grown in a Scienta Omicron EVO-50 MBE system, with a base pressure of 10^{-11} Torr. Commercially obtained (Shinkosha) $SrTiO_3$ (001) substrates were chemically etched to obtain atomic steps which included 45 minutes in deionized water at 85 ^oC and 45 minutes in 25% HCl at room temperature. Temperatures reported during growth were measured using a mid-infrared camera (Optris Xi 400) with an emissivity of 0.7. The substrate was outgassed for two hours at 600 ^oC prior to growth. During FeTe growth, high-purity Fe (99.99%) and Te (99.999%) elemental sources were simultaneously evaporated from thermal Knudsen effusion cells at a substrate temperature of 320° C. $ZrTe_2$ was grown at substrate temperatures ranging from $250-460$ ^oC. Zr (purity: grade 702, Kurt J.Lesker) was evaporated via e-beam. Reflection high-energy electron diffraction (RHEED) was monitored using a 13 keV electron beam during the growth of the samples.

All heterostructures studied in this paper start out with the growth of a 35 UC FeTe layer. We then deposit a $ZrTe_2$ film of varying thickness. In some samples, we follow this with a thin $CrTe_2$ film. To avoid degradation from oxidation upon *ex situ* exposure to ambient (a particular problem because of the presence of Zr), all samples are capped with 10 nm Te deposited at 10 ^oC.

II. TRANSMISSION ELECTRON MICROSCOPY

Prior to TEM sample preparation, the wafer was coated with approximately 15 nm of amorphous carbon using a Leica EM ACE200 sputter coater. Next, an FEI Helios 660 dual-beam focused ion beam (FIB)/scanning electron microscope (SEM) was utilized to create electron-transparent lamellae for TEM analysis, with SEM accelerating voltages and currents

* ski5160@psu.edu

† nxs16@psu.edu

varying from 3 to 5 kV/0.40 nA to 13 nA, and ion beam accelerating voltages and currents ranging from 2 to 30 kV/23 pA to 9.3 nA. HAADF-STEM images and energy dispersive X-ray spectroscopy (EDX) maps were obtained using a dual spherical aberration-corrected FEI Titan^3 G2 60-300 S/TEM at an accelerating voltage of 300 kV, a convergence angle of 25.2 mrad, and HAADF collection angles between 51 and 300 mrad.

III. X-RAY DIFFRACTION

XRD in Fig. 1 was measured in the Bragg-Brentano geometry on a Malvern Panalytical Empyrean system using Cu Kα_1 and Kα_2 radiation with iCore, dCore optics using $1/4^o$ slits, a Ni filter, and the detector in scanning-line mode with an active length of 3.347^o. Shifts in the FeTe peak positions from the bulk reference PDF #01-080-3499 are consistent with strain due to the lattice mismatch with the $SrTiO_3$ substrate. Additionally XRD ϕ-scans were performed on a Malvern Panalytical MRD 4-circle diffractometer using a parabolic mirror with a $1/4^o$ slit and the detector in receiving slit mode with an active length of 1 mm.

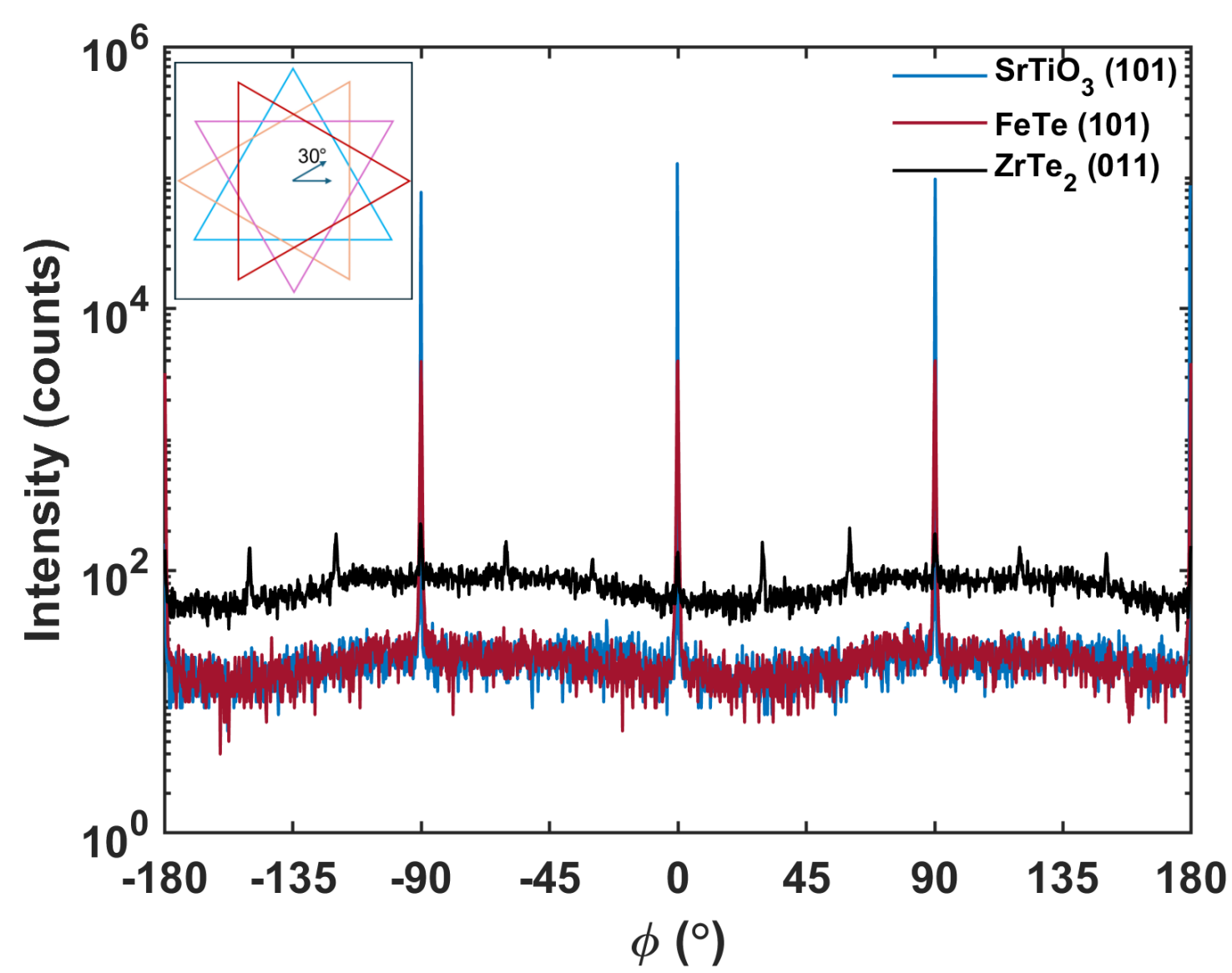

FIG. 1. XRD ϕ scans showing epitaxial relationship of $SrTiO_3$, FeTe and $ZrTe_2$. FeTe grows aligned to the $SrTiO_3$ substrate while the $ZrTe_2$ shows 30° twin domains, consistent with possible stackings of a trigonal material grown on cubic FeTe, as illustrated in the inset where each 30° rotated triangle maintains a face parallel to a face of the square representing the FeTe lattice.

IV. ANGLE RESOLVED PHOTO-EMISSION SPECTROSCOPY

The electronic band structure of the heterostructures is measured via *in-vacuo* transfer from the MBE chamber to an angle resolved photoemission spectroscopy (ARPES) chamber with excitation from the 21.2 eV spectral line of a helium plasma lamp. Photoemitted electrons are detected by a Scienta Omicron DA 30L analyzer with 6 meV energy resolution.

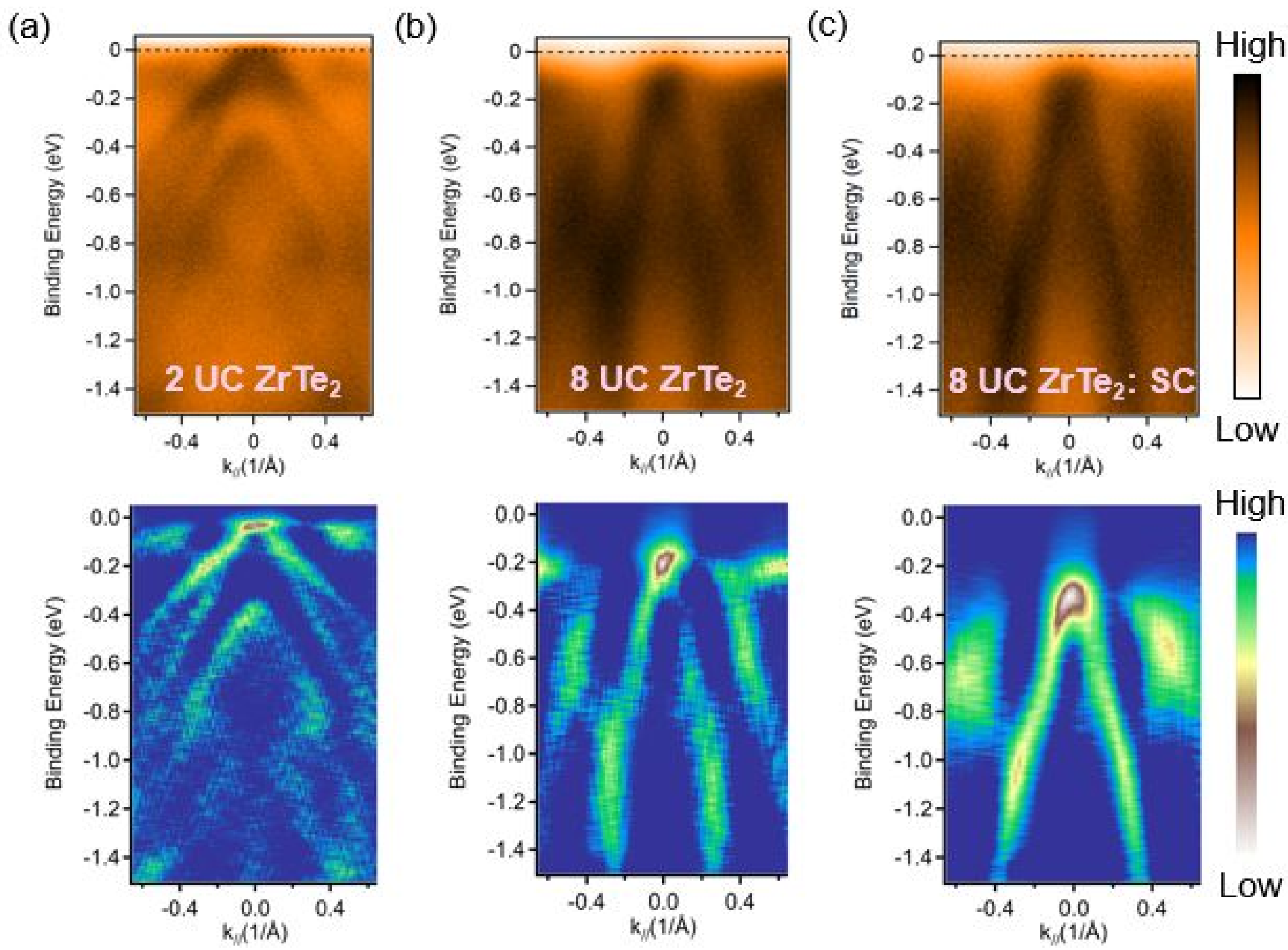

FIG. 2. Angle resolved photo-emission spectra (ARPES) of $ZrTe_2$/FeTe heterostructures grown using similar conditions for different $ZrTe_2$ thicknesses of (a) 2 UC (b) 8 UC and (c) 8 UC. Note that only the sample in (c) is confirmed to show a superconducting transition using electrical transport. The ARPES measurements are taken at $T = 300$ K along the $\bar{M} - \bar{\Gamma} - \bar{M}$ direction using 21.2 eV excitation from a helium lamp. The bottom panels show the second derivative of the spectra displayed in the corresponding top panel.

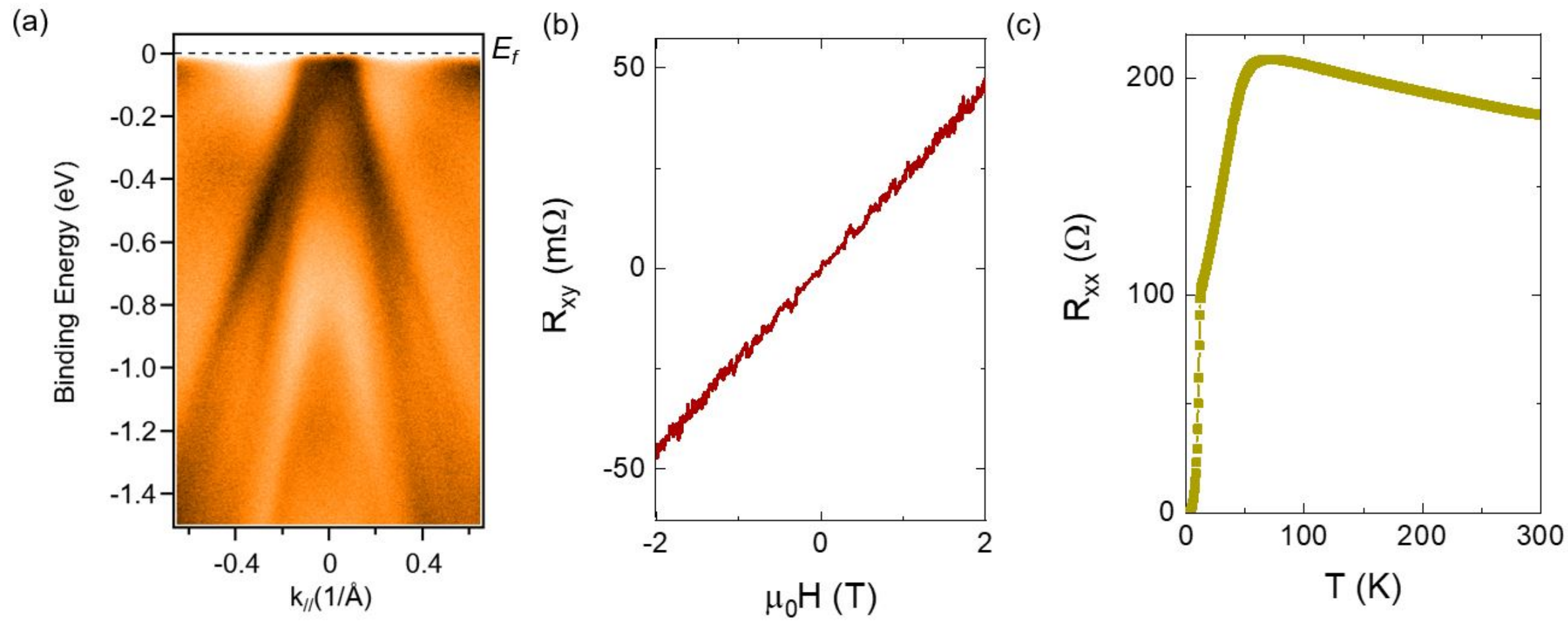

FIG. 3. Angle resolved photo-emission spectra (ARPES) of 6UC $ZrTe_2$/FeTe heterostructures measured at 300 K (b) Hall data measured at 300K with carrier density 5×10^{27} m^{-3}, consistent with hole-doping. (c) Resistance vs temperature of the same sample.

V. MAGNETIC FIELD DEPENDENCE OF DIFFERENT SAMPLES

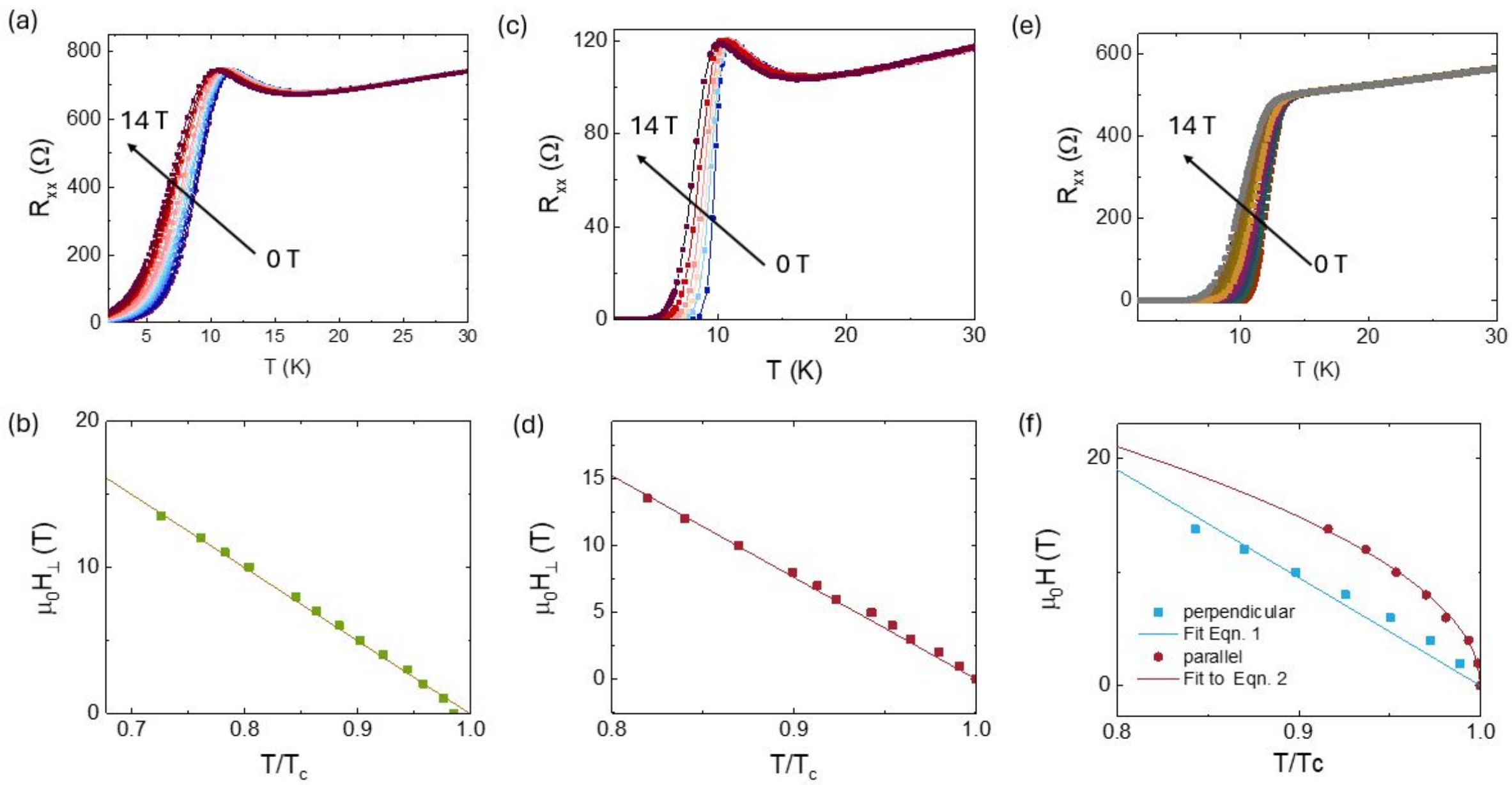

FIG. 4. Resistance vs temperature at different magnetic fields (a) 2UC $ZrTe_2$/35UC FeTe. (b) Fit to the magnetic field vs reduced temperature data, according to Ginsburg-Landau formalism. (c) 8UC $ZrTe_2$/35UC FeTe (d) Fit to the magnetic field vs reduced temperature data, according to Ginsburg-Landau formalism. (e) 1UC $CrTe_2$/6UC $ZrTe_2$/35 UC for fixed perpendicular magnetic field (f) Fit to the magnetic field vs reduced temperature data according to the Eq. 1 and Eq. 2 in the main text.

VI. TEMPERATURE DEPENDENCE OF CRITICAL CURRENT

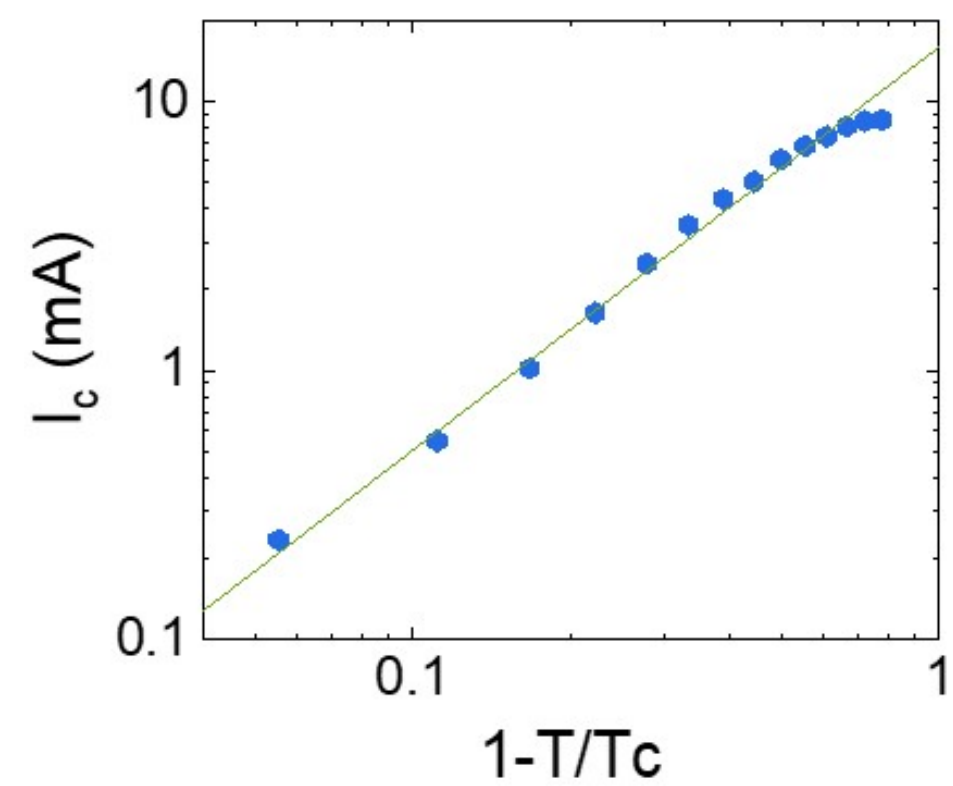

FIG. 5. The switching current density as a function of the reduced temperature follows Ginzburg-Landau power-law dependence $(1 - \frac{T}{T_c})^{3/2}$.

VII. SECOND HARMONIC MEASUREMENTS

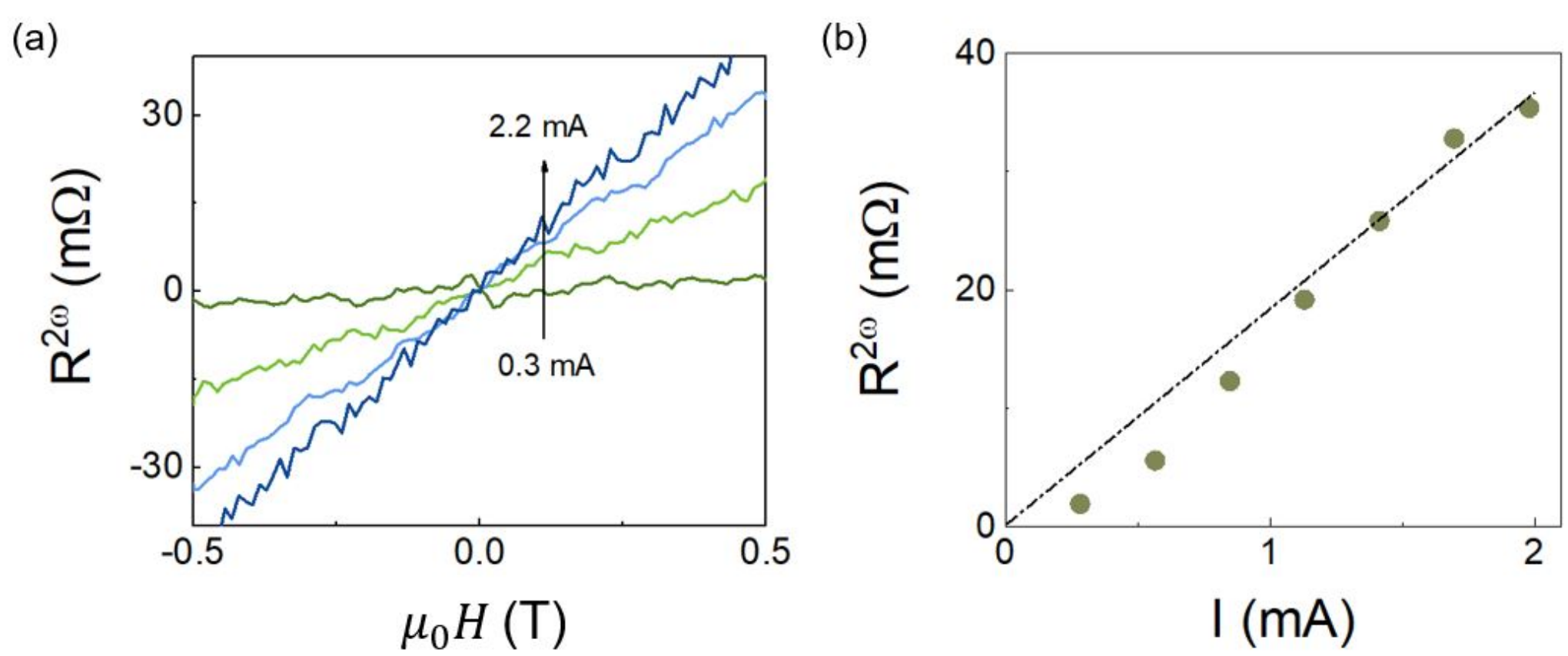

FIG. 6. (a) The magnetic-field dependence of the ratio of second harmonic resistance ($R^{2\omega}$) for different current values taken at $T = 10$ K. (b) The magnitude of $R^{2\omega}$ extracted at $\mu_0 H = 0.5$ T from the data shown in (a), varies linearly with the applied current.

VIII. ELECTRICAL PROPERTIES OF CRTE$_2$/ZRTE$_2$/FETE HYBRIDS

The resistive transitions and magneto-transport properties of 3UC $CrTe_2$/6UC $ZrTe_2$/35UC FeTe and 3UC $CrTe_2$/4UC $ZrTe_2$/35UC FeTe is shown in Fig. 7 and Fig. 8 respectively. We observe clear superconducting transition and hysteresis due to ferromagnetism at $T = 15$ K. This hysteresis survives till $T \approx 50$ K.

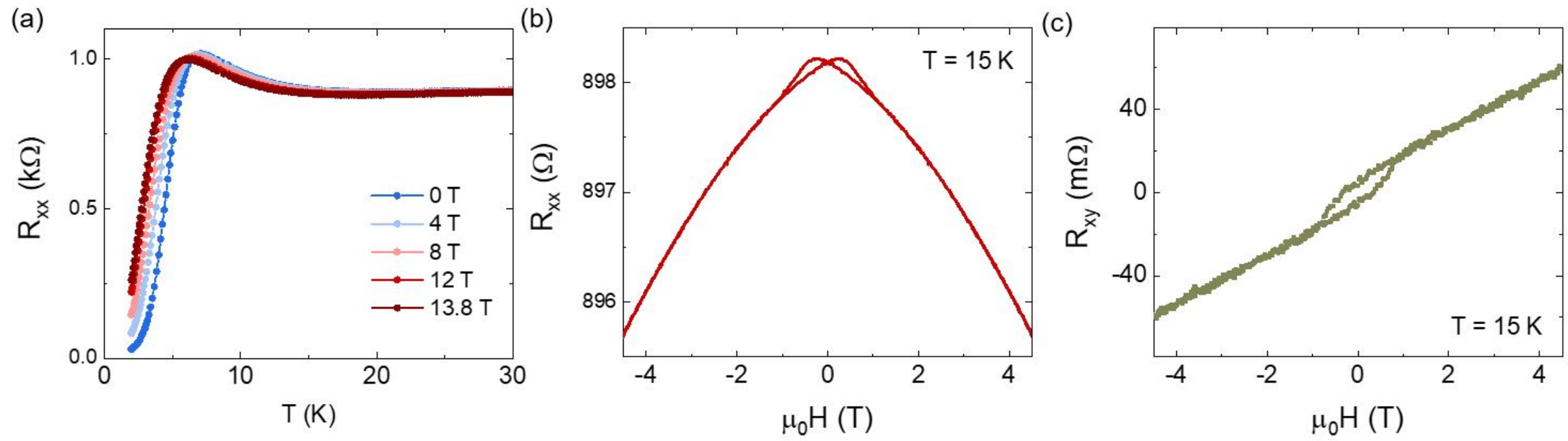

FIG. 7. (a) Resistance vs temperature at different magnetic fields (a) Longitudinal resistance (R_{xx}) vs. magnetic field. (c) Transverse resistance (R_{xy}) vs. magnetic field showing hysteresis.

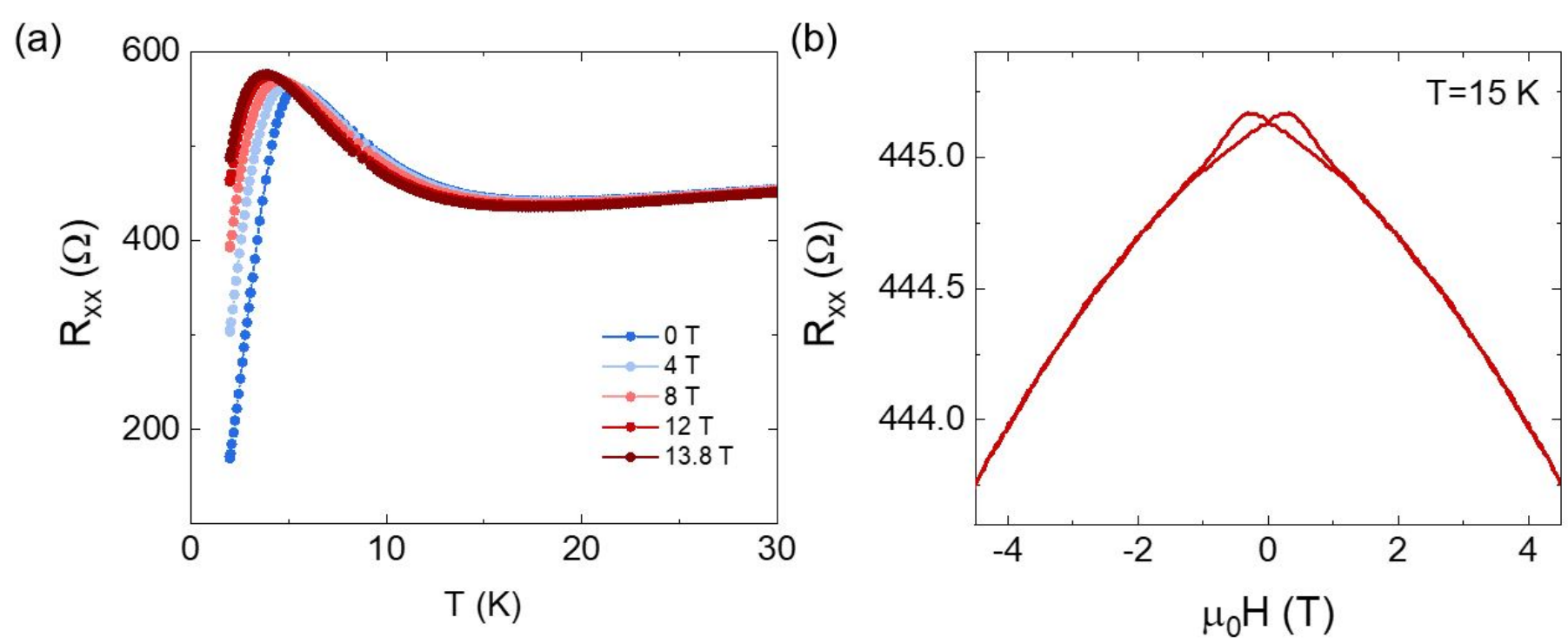

FIG. 8. (a) Resistance vs temperature at different magnetic fields (b) Longitudinal resistance (R_{xx}) vs. magnetic field.

IX. CURRENT-VOLTAGE CHARACTERISTICS

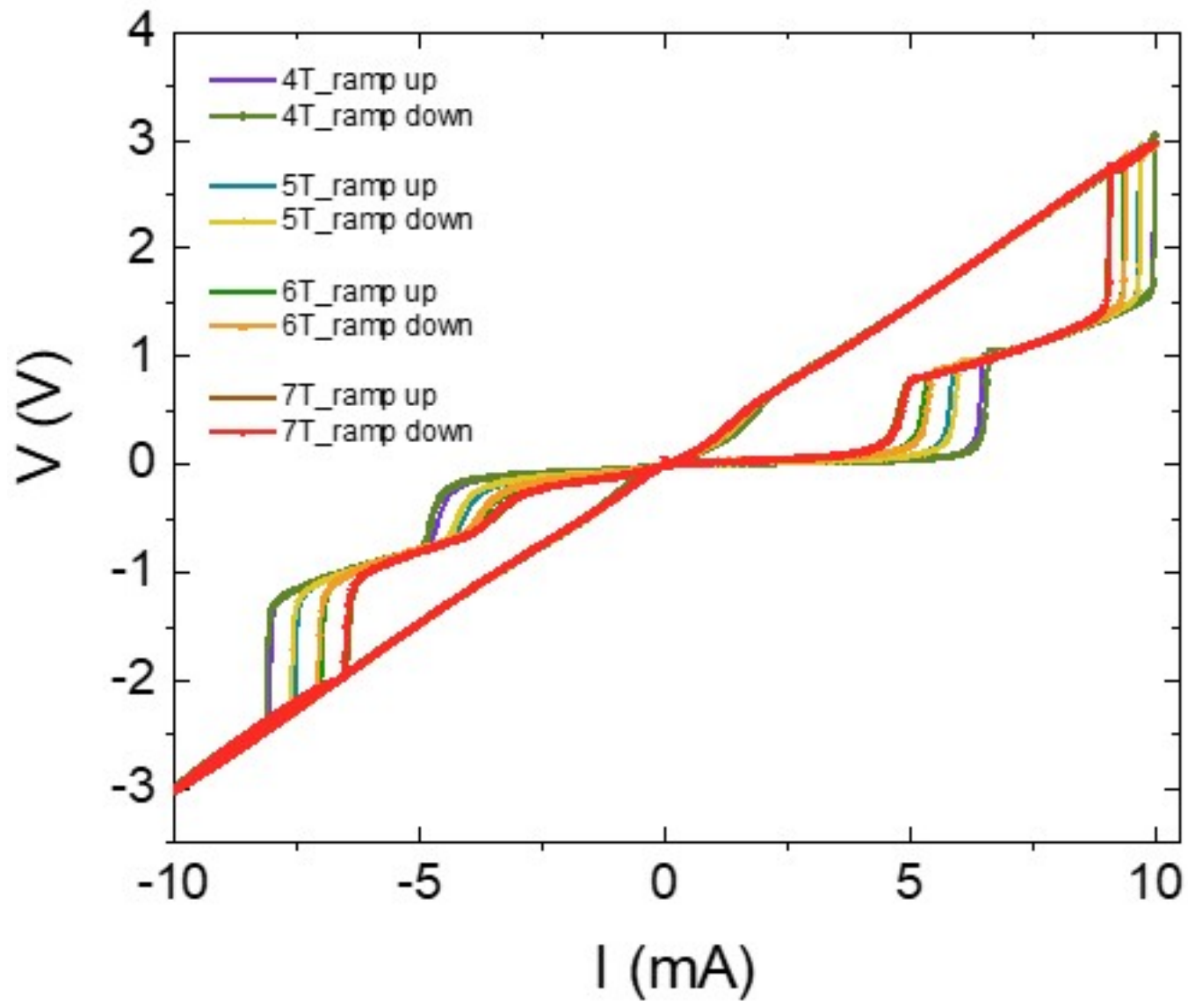

FIG. 9. (a) Current-voltage characteristics for 1 UC $CrTe_2$/6UC $ZrTe_2$/35UC at different magnetic fields showing reproducibility between sweeps.

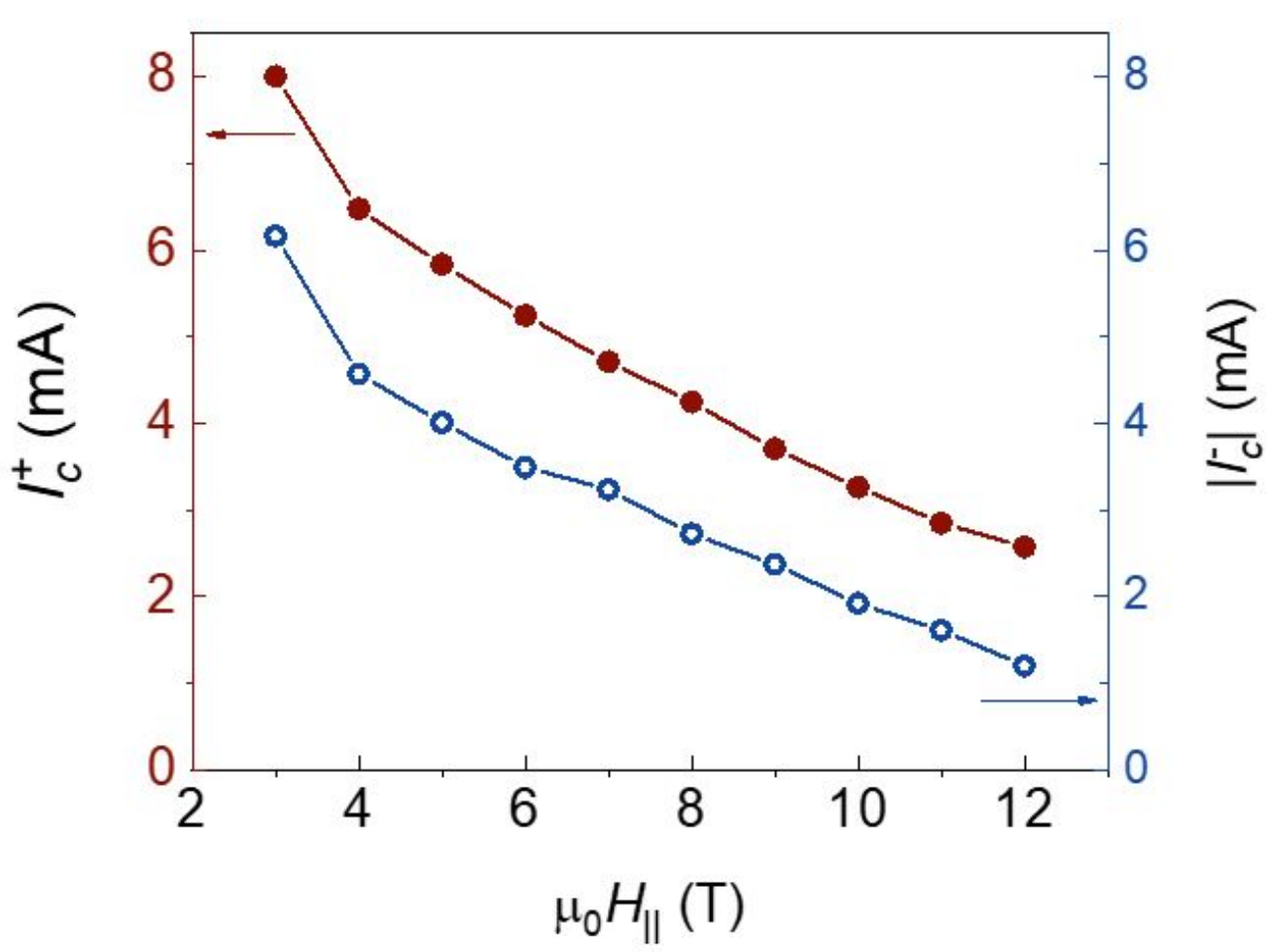

FIG. 10. (a) Critical current for positive (I_c^+, left axis) and negative ($|I_c^-|$, right axis) current sweep directions as a function of magnetic field

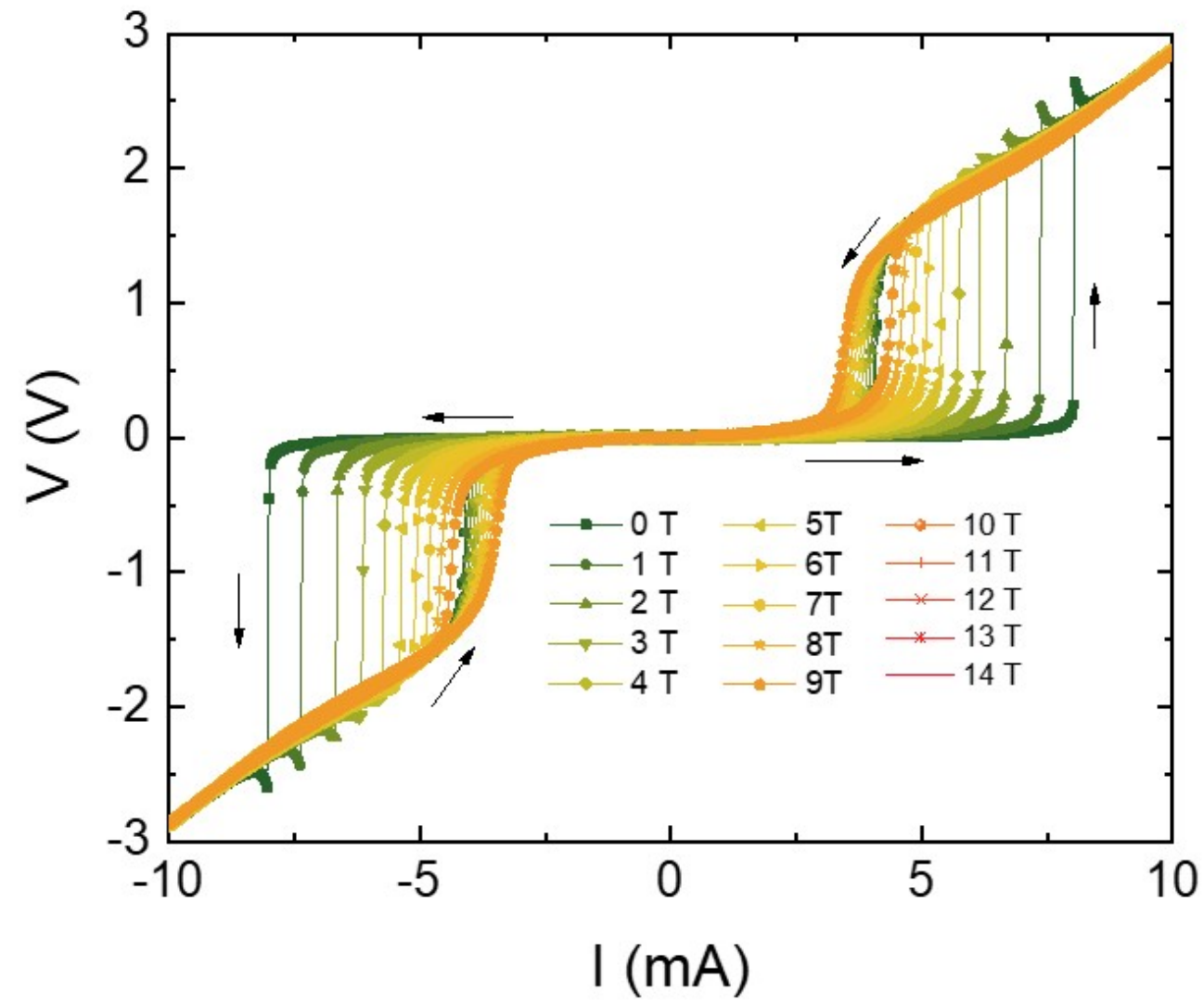

FIG. 11. (a) Current-voltage characteristics for 6UC $ZrTe_2$/35UC at different magnetic fields.

X. DFT CALCULATIONS

The first-principles DFT calculations were performed using Vienna ab initio simulation package (VASP) [1, 2]. A plane-wave basis set with a kinetic energy cutoff of 400 eV was employed to expand the electronic wavefunctions, and the projector-augmented wave (PAW) method was used to describe the pseudopotentials [3]. The exchange-correlation (XC) functional was treated within the generalized gradient approximation (GGA) of Perdew–Burke–Ernzerhof (PBE) [4].

The in-plane lattice constant of FeTe was fixed to be experimental value ($a_{FeTe} = 3.82$ Å). Then, we determined theoretical out-of-plane lattice constant of bulk FeTe by including Grimme D3 van der Waals correction [5], which method provides a reasonable interlayer distance ($a_{FeTe} = 6.229$ Å). The lattice constants of bulk $ZrTe_2$ were theoretically determined to be $a_{ZrTe_2} = 3.946$ Å and $c_{ZrTe_2} = 6.649$ Å. Then, we constructed 2L FeTe/2L $ZrTe_2$ heterostructure using a 4×1 FeTe supercell and a $2\sqrt{3} \times 1$ $ZrTe_2$ supercell. The in-plane lattice constants of the FeTe/$ZrTe_2$ heterostructure were determined to be $a = 3.885$ Å and $c = 14.4816$ Å, respectively. To evaluate relative stabilities of bicollinear and stripe AFM phases of 2L FeTe, we used a 2×2 magnetic supercell structure. The $18 \times 18 \times 12$, $12 \times 3 \times 1$ and $9 \times 9 \times 1$ k-grid samples were used for bulk FeTe ($ZrTe_2$), FeTe/$ZrTe_2$ heterostructure and 2×2 magnetic supercell of FeTe, respectively. Spin–orbit coupling was included in all

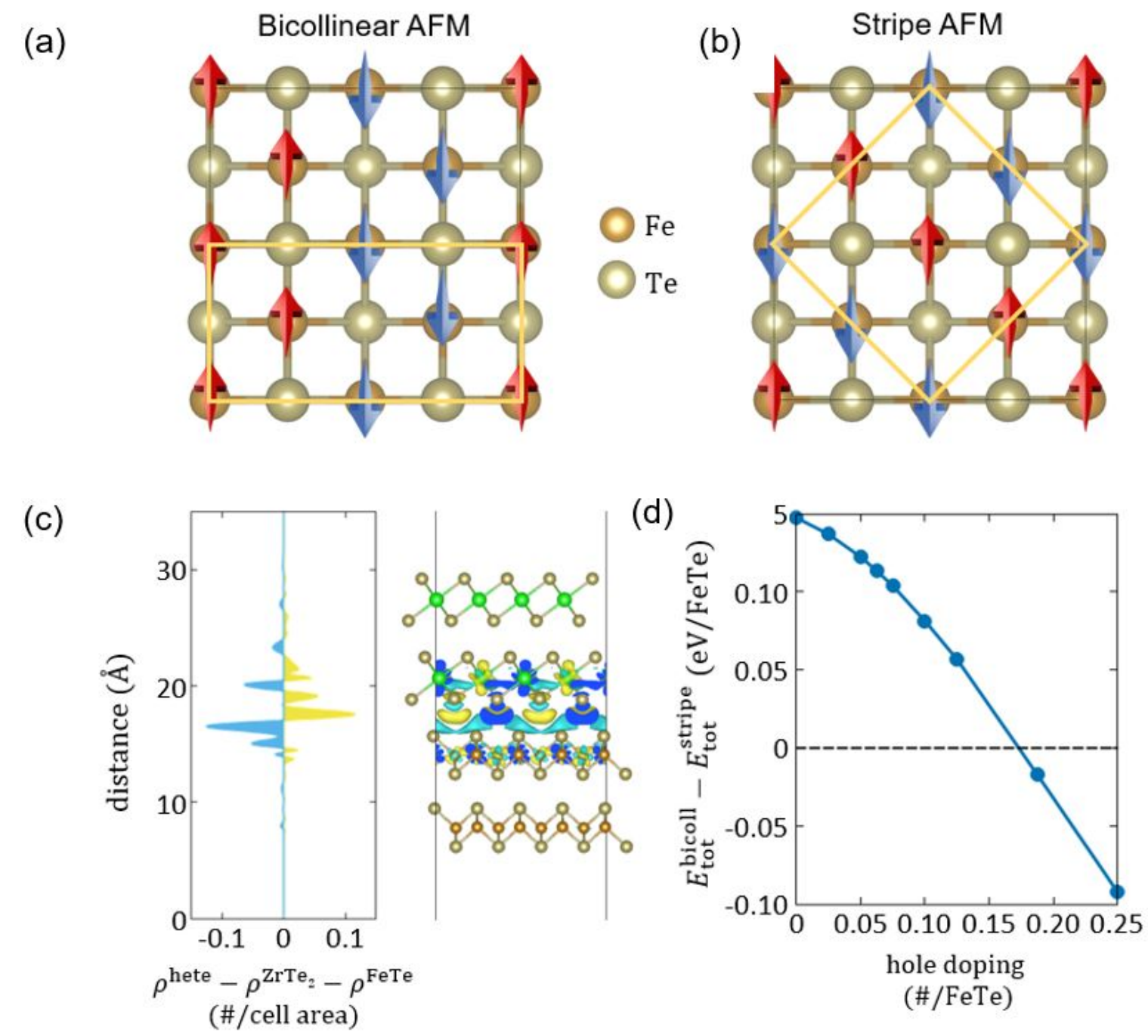

FIG. 12. Schematic diagrams of the (a) bicollinear antiferromagnetic (AFM) and (b) stripe AFM spin configurations of FeTe. (c) Plane-averaged charge transfer (left) and spatial-resolved transferred-charge distribution (right) of FeTe/$ZrTe_2$ heterostructure. (d) Relative total energies between bicollinear AFM (E_{tot}^{bicoll}) and stripe AFM (E_{tot}^{AFM}) of the bilayer FeTe as a function of hole doping.

electronic structure calculations.

As a first step, we investigate the role of the $ZrTe_2$ layer by calculating the interfacial charge transfer in $ZrTe_2$/FeTe heterostructures (Fig. 12(c)). The results reveal an accumulation of electrons in the $ZrTe_2$ layer, indicating hole doping in FeTe near the interface. This finding is further supported by additional DFT calculations for isolated bilayer $ZrTe_2$ and FeTe, which show that 2 UC $ZrTe_2$ has a higher work function (4.83 eV) than 2 UC FeTe (4.25 eV). Next, we examine the relative stability of the bicollinear and stripe antiferromagnetic phases in 2 UC FeTe as a function of hole doping (Fig. 12(d)). In the pristine case, although the bicollinear AFM is the experimental ground state of FeTe, the total energy of the stripe AFM phase is calculated to be lower than that of the bicollinear AFM, consistent with previous theoretical reports [6]. We note that the relative stability of magnetic phases can be sensitive to various computational details.

To further investigate the role of the $CrTe_2$ capping layer, we calculated the magnetic proximity in a 1L ferromagnetic $CrTe_2$/6L $ZrTe_2$ heterostructure. The in-plane lattice constant of the heterostructure was chosen as 3.799 Å, the average of the lattice constants of isolated 1L $CrTe_2$ and bulk $ZrTe_2$. This choice imposes +4.02% tensile strain on the $CrTe_2$ monolayer and -3.72% compressive strain on the $ZrTe_2$ layers. The Brillouin zone of the heterostructure was sampled with a $18 \times 18 \times 1$ k-point mesh.

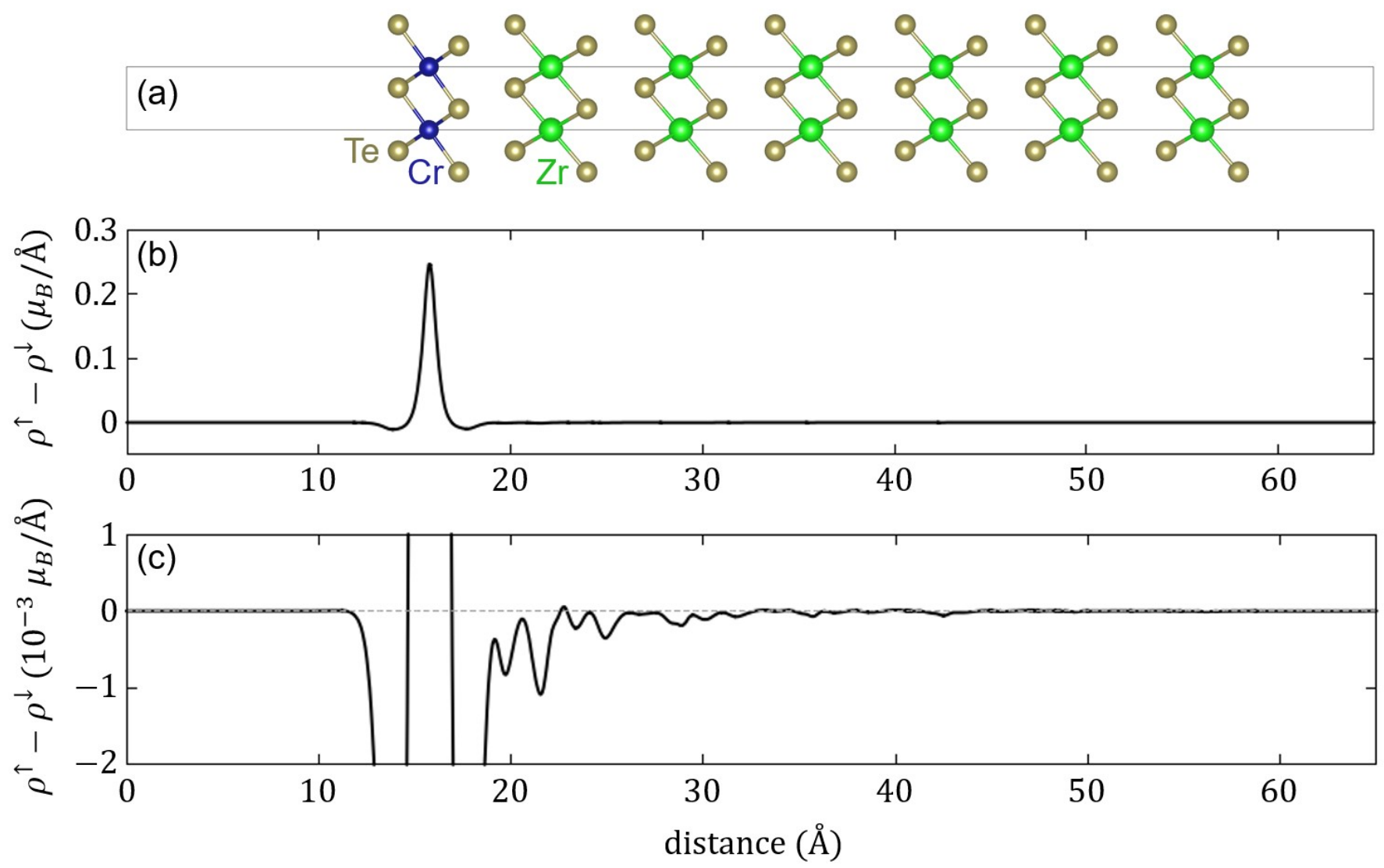

FIG. 13. (a) Crystal structure of 1L $CrTe_2$/ 6L $ZrTe_2$ heterostructure (b) Planar-averaged spin density $\rho^{\uparrow} - \rho^{\downarrow}$ along the out-of-plane direction and (c) its magnified view (10^{-3} μ_B/Å scale).

A. Additional Note

Yuan and Fu [7] write the GL free energy for a single-q condensate as

$$F(\vec{q}, \Delta) = \alpha_q|\Delta|^2 + \frac{\beta}{2}|\Delta|^4 \tag{1}$$

They then show that for a Rashba superconductor under a weak in-plane Zeeman field B,

$$\alpha_q = t - \left(b_0 - b_1 q^2\right) \vec{q} \cdot \left(\vec{B} \times \hat{z}\right) + a_0 q^2 \tag{2}$$

Here, Δ is the superconducting order parameter and $\vec{q}$ is the center-of-mass momentum of the Cooper pairs, equivalently the condensate phase gradient associated with a supercurrent.

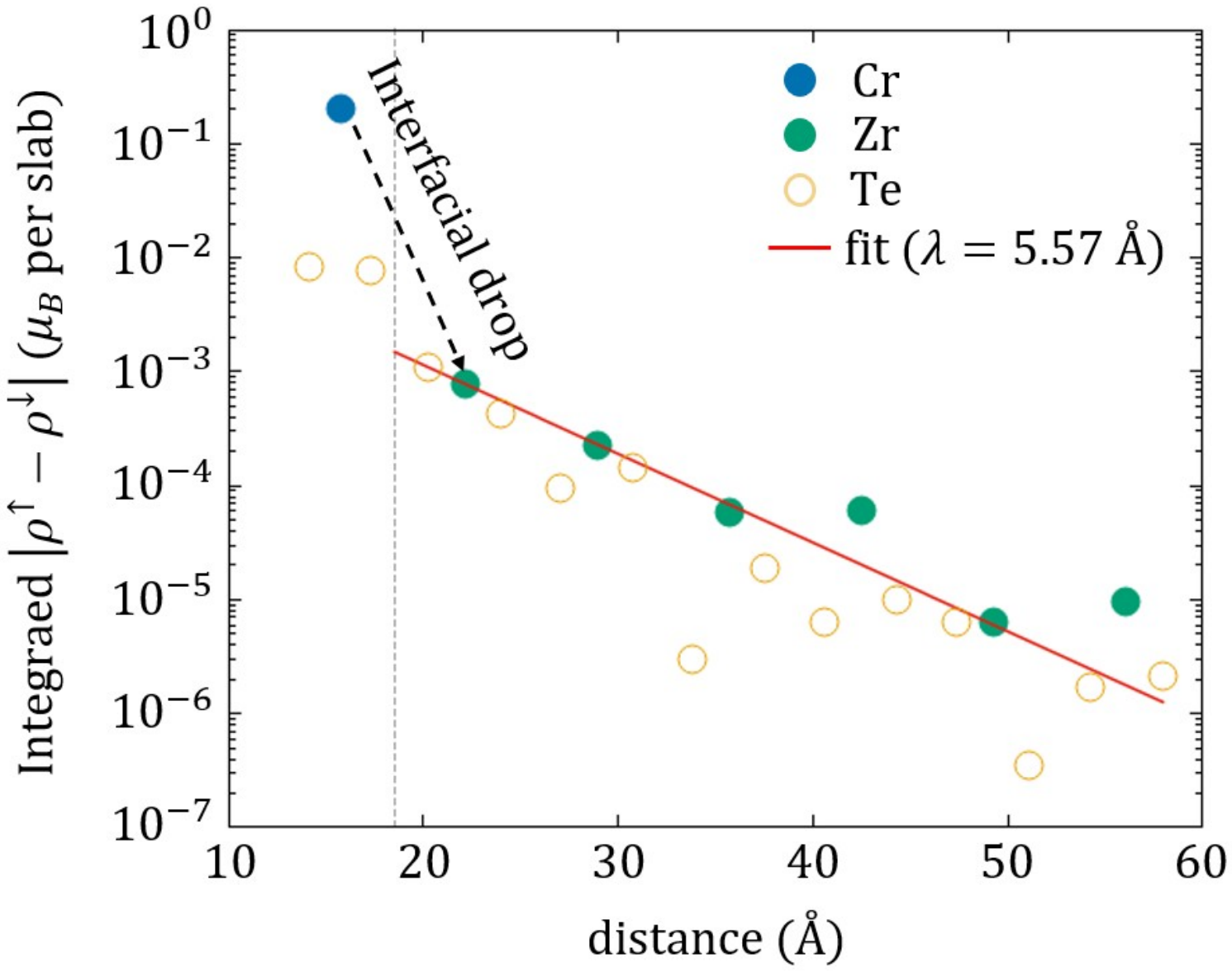

FIG. 14. Spatial decay of the proximity-induced spin polarization across the heterostructure. The integrated magnetization $|\rho^{\uparrow}-\rho^{\downarrow}|$ per slab is plotted on a logarithmic scale against distance from the $CrTe_2$ layer, resolved by atomic species (Cr, Zr, Te). A sharp interfacial drop separates the magnetic $CrTe_2$ layer from the $ZrTe_2$ region, where the spin decays exponentially with a characteristic decay length of $\lambda = 5.57$ Å (red fit).

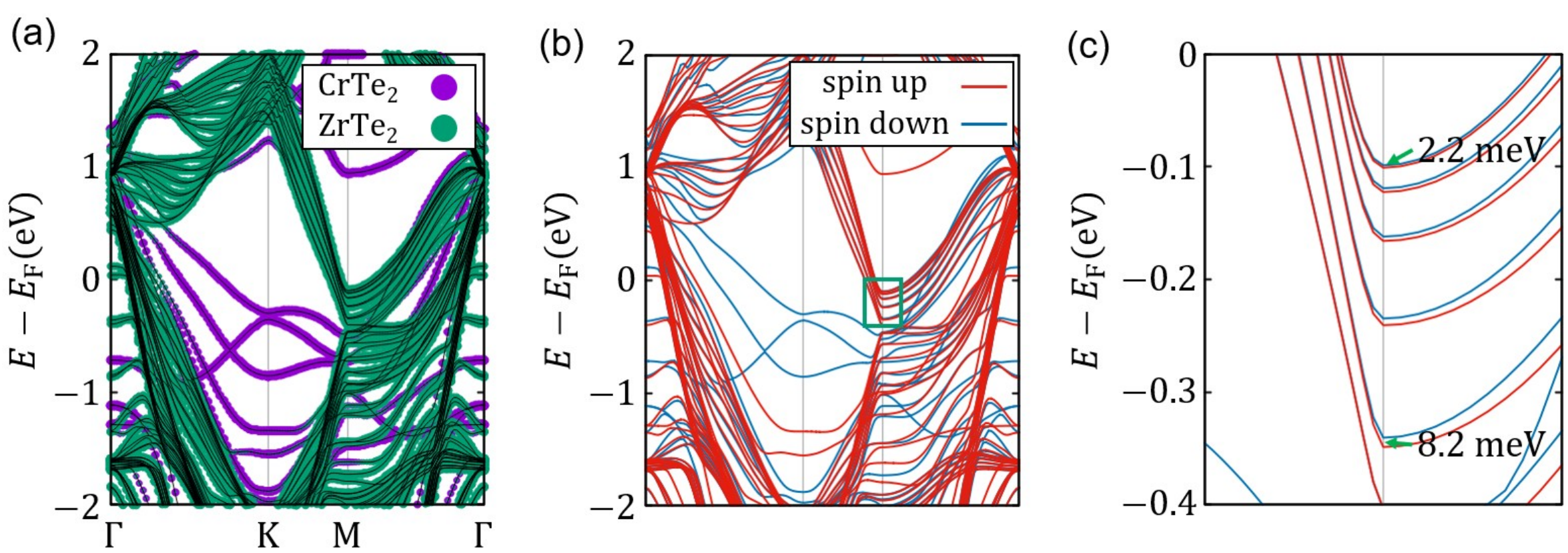

FIG. 15. Electronic band structure of the $ZrTe_2/CrTe_2$ heterostructure. (a) Orbital-projected bands showing the contributions of $CrTe_2$ (purple) and $ZrTe_2$ (green). (b) Spin-resolved band structure, with spin-up (red) and spin-down (blue) channels; the green box marks the region of interest near the Fermi level. (c) Magnified view of the boxed region, resolving spin-split bands with splittings of 2.2 8.2 meV.

The coefficient α_q is the quadratic Ginzburg-Landau coefficient for a superconducting state with momentum $\vec{q}$, while β is the quartic coefficient that stabilizes the superconducting free energy. The parameter t measures the distance from the superconducting transition, a_0 is the conventional positive phase-stiffness or gradient-energy coefficient, and b_0 and b_1 parameterize the odd-in- terms allowed by Rashba spin-orbit coupling in the presence of an in-plane Zeeman field B. The unit vector $\hat{z}$ denotes the polar axis normal to the two-dimensional interface, so $\vec{q} \cdot \left(\vec{B} \times \hat{z}\right)$ selects the component of the condensate momentum along the field-induced Rashba helical direction.

This is exactly the structure we want: a quadratic term, a linear-in-q term, and a cubic odd-in-q term. Yuan and Fu explicitly state that odd-power derivative terms are allowed in 2D noncentrosymmetric superconductors under a parallel magnetic field, and that the third-order term skews α_q and produces different critical currents in the opposite directions. So the more faithful equation would be written as

$$F(\vec{q}, \Delta) = \left\{t + a_0 q^2 - b_0\, \vec{q} \cdot (\vec{h}_{\rm eff} \times \hat{z}) + b_1 q^2 \vec{q} \cdot (\vec{h}_{\rm eff} \times \hat{z})\right\} |\Delta|^2 + \frac{\beta}{2} |\Delta|^4 \tag{3}$$

This maps directly onto Yuan and Fu by the replacements of the magnetic field $\vec{B}$ with the appropriate generalization in our heterostructure

$$\vec{h}_{\rm eff} = \vec{h}_{\rm ex} + \vec{h}_{\rm prox} \tag{4}$$

that due to an applied external magnetic field and a proximity exchange field due to the $CrTe_2$.

XI. EFFECT OF GROWTH TEMPERATURE

The resistive transitions obtained in different samples as the growth temperature of $ZrTe_2$ is varied are shown in Fig. 16.

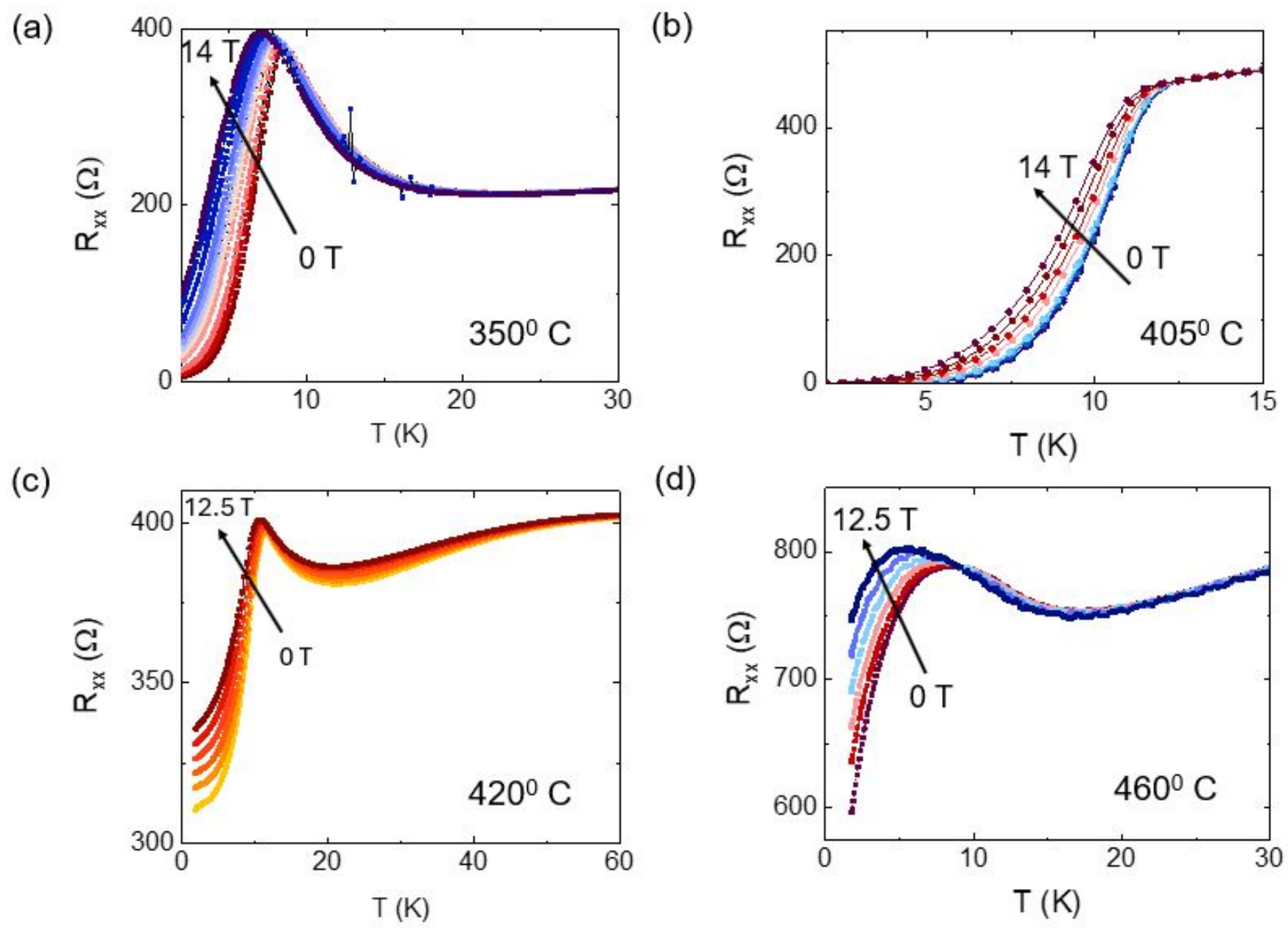

FIG. 16. Resistance vs temperature for (a) 8UC $ZrTe_2$ at 350 ^{0}C (b) 8UC $ZrTe_2$ at 405 ^{0}C (c) 8UC $ZrTe_2$ at 420 ^{0}C (d) 6UC $ZrTe_2$ at 460 ^{0}C.

---

[1] Kresse, G.; Hafner, J. Ab initio molecular dynamics for liquid metals. *Phys. Rev. B* **1993**, *47*, 558.

[2] Kresse, G.; Furthmüller, J. Efficient iterative schemes for ab initio total-energy calculations using a plane-wave basis set. *Phys. Rev. B* **1996**, *54*, 11169.

[3] Kresse, G.; Joubert, D. From ultrasoft pseudopotentials to the projector augmented-wave method. *Phys. Rev. B* **1999**, *59*, 1758.

[4] Perdew, J. P.; Burke, K.; Ernzerhof, M. Generalized gradient approximation made simple. *Phys. Rev. Lett.* **1996**, *77*, 3865.

[5] Grimme, S.; Antony, J.; Ehrlich, S.; Krieg, H. A consistent and accurate ab initio parametrization of density functional dispersion correction (DFT-D) for the 94 elements H-Pu. *J. Chem. Phys.* **2010**, *132*.

[6] Koteski, V.; Ivanovski, V. N.; Umićević, A.; Belošević-Čavor, J.; Toprek, D.; Mahnke, H.-E. First-principles calculations of tetragonal FeX (X= S, Se, Te): Magnetism, hyperfine-interaction, and bonding. *J. Magn. Magn. Mater* **2017**, *441*, 769–775.

[7] Yuan, N. F.; Fu, L. Supercurrent diode effect and finite-momentum superconductors. *Proc. Nat. Acad. Sci.* **2022**, *119*, e2119548119.
